# New Oxidized Zoanthamines from a Canary Islands *Zoanthus* sp.

**DOI:** 10.3390/md12105188

**Published:** 2014-10-14

**Authors:** Francisco Cen-Pacheco, Manuel Norte Martín, José Javier Fernández, Antonio Hernández Daranas

**Affiliations:** 1 Institute of Bio-Organic Chemistry “Antonio González”, Center for Biomedical Research of the Canary Islands, University of La Laguna, 38206 Tenerife, Spain; E-Mails: fcen@uv.mx (F.C.); mnorte@ull.es (M.N.M.); 2 Faculty of Bioanalysis Campus-Veracruz, Universidad Veracruzana, 91700 Veracruz, Mexico; 3 Department of Organic Chemistry, Faculty of Health Sciences, University of La Laguna, 38206 Tenerife, Spain; 4 Department of Chemical Engineering and Pharmaceutical Technology, Faculty of Health Sciences, University of La Laguna, 38206 Tenerife, Spain

**Keywords:** *Zoanthus*, zoanthamine alkaloids, marine natural products

## Abstract

Three new norzoanthamine-type alkaloids, named 2-hydroxy-11-ketonorzoanthamide B (**1**), norzoanthamide B (**2**) and 15-hydroxynorzoanthamine (**3**), were isolated from *Zoanthus sp.* specimens collected at the Canary Islands. Their structures were determined by interpretation of NMR and HR-ESIMS data. Relative configurations of their chiral centers were proposed on the basis of ROESY spectra and by comparison of their spectroscopic data with those of the well-known compound, norzoanthamine.

## 1. Introduction

Marine zoanthids are widely dispersed throughout the temperate and tropical littoral regions of the Indic, Pacific and Atlantic Oceans. Diverse species of these organisms produce a wide array of metabolites, some of which have unique structures and possess significant biological activities [1]. In particular, from the genus *Zoanthus* (phylum Cnidaria, class Anthozoa, order Zoanthidea), different research groups have isolated a series of alkaloids, known as zoanthamines, characterized by a unique polycyclic backbone [2]. Zoanthamine, isolated in 1984 from polyps collected at the Visakhapatnam coast of India by Faulkner *et al.* [3], was the first example of these compounds. Afterwards, norzoanthamine was identified from a colony of polyps collected at the Ayamaru coast of the Amami Island, South Japan, by Uemura and co-workers [4]. Later, other research groups reported a significant number of related compounds, some of which have substantial structural modifications [5,6,7,8,9]. From a pharmacological point of view, different biological effects, including anti-inflammatory, antiplatelet and antiosteoporotic activities, have been investigated [10,11,12]. In particular, the increasing worldwide prevalence of osteoporosis and the novel mechanism of action of zoanthamines have set them up as promising drug candidates [13]. Accordingly, a number of synthetic approaches to obtain these molecules independently from organism collection have been published [14,15,16].

**Figure 1:**
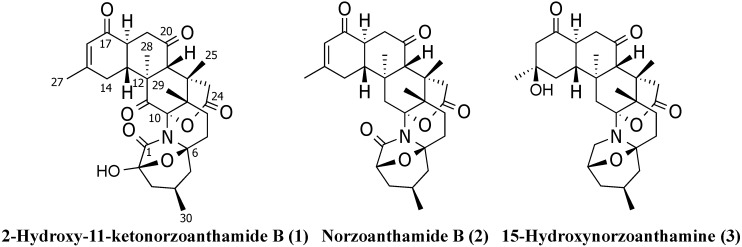
Structures of three new zoanthamine-type alkaloids isolated from *Zoanthus* sp.

In this paper, we describe the isolation and structural characterization of three new oxidized norzoanthamine congeners from the genus *Zoanthus* collected at the coast of Tenerife. The structures of these new metabolites, 2-hydroxy-11-ketonorzoanthamide B (**1**), norzoanthamide B (**2**) and 15-hydroxynorzoanthamine (**3**), were determined primarily on the basis of MS and NMR data (Figure 1).

## 2. Results and Discussion

**Figure 2:**
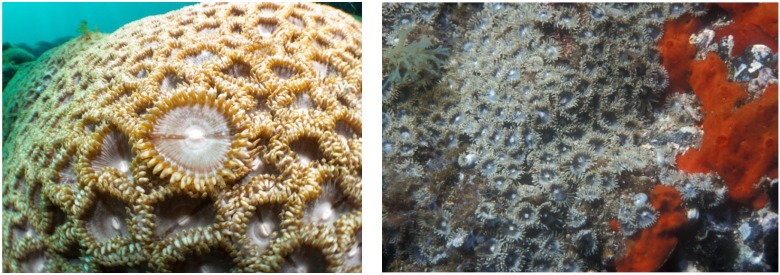
Colonies of *Zoanthus* sp. (Photos courtesy of Alberto Brito).

Colonies of *Zoanthus* sp. were collected by hand in the intertidal zone of Punta del Hidalgo, Tenerife, Canary Islands (28°34′35.06ʺ N; 16°19′43.64ʺ W) (Figure 2). Fresh specimens (120 g) were extracted with MeOH (3 × 2 L each time) at room temperature and the solvent removed *in vacuo* to give a brownish viscous oil (1.9 g). The extract was chromatographed on a Sephadex LH-20 column (3-cm internal diameter and 20-cm length) using MeOH and collecting 60 tubes of 10 mL. A second chromatographic step included a Lobar LiChroprep-RP18 column using MeOH:H_2_O (7:3) as the eluent at 2 mL/min flux. Two fractions, obtained between 8–16 min and 17–25 min, were further purified by HPLC using a μ-Bondapack C-18 column and CH_3_CN:MeOH:H_2_O (2:1:1) or (2:1:2), respectively, as mobile phases at 2 mL/min. This procedure yielded 0.50 mg of 2-hydroxy-11-ketonorzoanthamide B (**1**), 0.45 mg of norzoanthamide B (**2**) and 0.30 mg of 15-hydroxynorzoanthamine (**3**), together with the previously known norzoanthamine, zoaramine and zoarenone [9].

2-Hydroxy-11-ketonorzoanthamide B (**1**) was isolated as an optically active powder [α]^25^_D_ +9 (*c* 0.045, CHCl_3_). Its molecular formula was established by ESI-HRMS as C_29_H_35_NO_8_ (*m*/*z* 526.24340; calculated for 526.24354, C_29_H_36_NO_8_, [M + H]^+^), requiring thirteen degrees of unsaturation. The ^1^H NMR spectrum of **1** showed a number of signals characteristic norzoanthamine, including four methyl singlets at δ_H_ 1.01, 1.03, 1.34 and 2.01, one methyl doublet at δ_H_ 1.00, the AX system corresponding to CH_2_-23 (δ_H_ 2.55 and 4.27, *J* = 20.6 Hz), as well as the distinctive olefinic methine proton at δ_H_ 5.90 (s). Analysis of the HSQC and HMBC experiments allowed us to classify the existent 29 carbon signals as five methyls, seven methylenes, five methines and twelve quaternary carbons, including two carboxylic and three carbonyl carbons at δ_C_ 168.1, 170.6, 195.8, 197.9 and 206.9 (Table 1).

**Figure 3:**
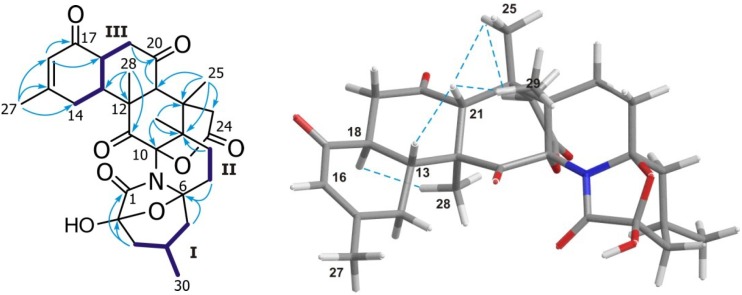
(**Left**) HMBC correlations for 2-hydroxy-11-ketonoroanthamide B (**1**) are indicated by arrows. ^1^H-^1^H spin systems **I**–**III** are showed in bold blue lines; (**Right**) significant dipolar correlations observed in the ROESY spectrum are indicated by a dotted blue line.

Analysis of the COSY spectrum revealed the presence of three isolated ^1^H-^1^H spin-systems (**I**–**III** as showed in Figure 3). The first system (Fragment **I**) includes methyl CH_3_-30 (δ_H_ 1.00, d, *J* = 6.0 Hz; δ_C_ 20.9), which is coupled with CH-4 (δ_H_ 2.05; δ_C_ 24.5) and this, in turn, with both CH_2_-3 (δ_H_ 1.43/2.03; δ_C_ 38.0) and CH_2_-5 (δ_H_ 1.15/2.05; δ_C_40.3). Fragment **II** includes only methylenes CH_2_-7 (δ_H_ 1.92/2.14; δ_C_ 28.7) and CH_2_-8 (δ_H_ 1.70/1.86; δ_C_ 23.8). Finally, Fragment **III** was started from methylene CH_2_-14 (δ_H_ 2.17/2.98; δ_C_ 32.7) that is coupled with CH-13 (δ_H_ 2.76; δ_C_ 46.7), in turn, connected with CH-18 (δ_H_ 2.70; δ_C_ 46.0) and CH_2_-19 (δ_H_ 2.48/2.67; δ_C_ 42.7) (Figure 3). The significant number of quaternary carbons existent within this molecule made the HMBC experiment an essential tool to connect them with the previously described ^1^H-^1^H spin-systems. Thus, HMBC correlations of H_2_-3 with carbon C-1 (δ_C_ 170.6) and C-2 (δ_C_ 100.6), together with the cross-peaks of H_2_-5 and H_2_-7 with C-6 (δ_C_ 90.2), allowed us to establish a seven-membered lactam ring. The chemical shifts of C-2 and C-6 indicated that both are ketal carbons and allowed us to build the azabicycle [3.2.1] system present in this new metabolite. In addition, the HMBC correlations observed from methines H-16 (δ_H_ 5.90, s) and H-21 (δ_H_ 2.89, s), as well as those from methyls CH_3_-25, CH_3_-27, CH_3_-28 and CH_3_-29 resulted in it being very valuable to build the polycyclic core of the molecule (Figure 3).

The relative configuration of **1** was deduced by analysis of the ROESY spectrum and ^1^H-^1^H coupling constants values together with a comparison of its chemical shift values with those of norzoanthamine. Particularly important were the dipolar correlations between H-21 (δ_H_ 2.89) and H-13 (δ_H_ 2.76), H_3_-25 (δ_H_ 1.01) and H_3_-29 (δ_H_ 1.03) together with those of H-18 (δ_H_ 2.70) and H_3_-28 (δ_H_ 1.34), which allowed us to propose a 2R*, 4R*, 6S*, 9S*, 10S*, 12S*, 13R*, 18S*, 21R*, 22S*, 23S* relative configuration for **1** (Figure 3).

**Table 1:** NMR data (CDCl_3_) for 2-hydroxy-11-ketonorzoanthamide B **(1**).

2-Hydroxy-11-ketonorzoanthamide B (1)							
C	δ ^13^C	δ ^1^H	*J* (Hz)	C	δ ^13^C	δ^1^H	*J* (Hz)
**1**	170.6, C	-	-	**16**	124.5, CH	5.90	s
**2**	100.6, C	-	-	**17**	197.9, C	-	-
**3**	38.0, CH_2_	1.43 (β); 2.03 (α)	11.2; 12.0 m	**18**	46.0, CH	2.70	5.8; 11.3; 12.0
**4**	24.5, CH	2.05	m	**19**	42.7, CH_2_	2.48 (β); 2.67 (α)	11.3; 13.3; 5.8; 13.3
**5**	40.3, CH_2_	1.15 (β); 2.05 (α)	12.3; 14.8 m	**20**	206.9, C	-	-
**6**	90.2, C	-	-	**21**	60.8, CH	2.89	s
**7**	28.7, CH_2_	1.92 (α); 2.14 (β)	2.9; 4.0; 13.7; 3.1; 12.1; 13.7	**22**	38.1, C	-	-
**8**	23.8, CH_2_	1.70 (β); 1.86 (α)	3.1; 4.0; 13.4; 2.9; 12.1; 13.4	**23**	34.6, CH_2_	2.55 (β); 4.27 (α)	20.6; 20.6
**9**	43.9, C	-	-	**24**	168.1, C	-	-
**10**	95.7, C	-	-	**25**	20.9, CH_3_	1.01	s
**11**	195.8, C	-	-	**27**	24.4, CH_3_	2.01	s
**12**	54.9, C	-	-	**28**	14.1, CH_3_	1.34	s
**13**	46.7, CH	2.76	3.6; 10.3; 12.0	**29**	17.7, CH_3_	1.03	s
**14**	32.7, CH_2_	2.17 (β); 2.98 (α)	10.3; 18.2; 3.6; 18.2	**30**	20.9, CH_3_	1.00	6.0
**15**	162.2, C	-	-				

**Table 2:** NMR data (CDCl_3_) for norzoanthamide B (**2**) and 15-hydroxynorzoanthamine (**3**).

C	Norzoanthamide B (2)			15-Hydroxynorzoanthamine (3)		
	δ ^13^C	δ^1^H	*J* (Hz)	δ ^13^C	δ^1^H	*J* (Hz)
**1**	175.0, C	-	-	47.1, CH_2_	3.23; 3.25	m
**2**	77.0, CH	4.25	2.1; 3.6	74.2, CH	4.55	m
**3**	32.7, CH_2_	1.41 (β); 1.86 (α)	3.6; 12.0; 13.5; 2.1; 4.5; 13.5	38.8, CH_2_	1.46; 1.55	m
**4**	23.8, CH	2.22	4.2; 4.5; 11.1; 12.0	22.9, CH	2.27	6.0; 11.1
**5**	40.8, CH_2_	1.18 (β); 2.19 (α)	11.1; 13.2; 4.2; 13.2	44.4, CH_2_	1.08 (β); 2.08 (α)	11.1; 14.0; 6.0; 14.0
**6**	93.4, C	-	-	89.9, C	-	-
**7**	29.8, CH_2_	1.90 (α); 2.08 (β)	3.1; 4.2; 13.5; 5.6; 13.3; 13.5	29.9, CH_2_	1.76; 1.89	m
**8**	22.6, CH_2_	1.63; 1.65	m	23.7, CH_2_	1.55; 1.67	m
**9**	39.8, C	-	-	39.9, C	-	-
**10**	96.6, C	-	-	102.0, C	-	-
**11**	40.2, CH_2_	2.30; 3.52	15.2; 15.2	42.1, CH_2_	1.90 (β); 2.13 (α)	14.0; 14.0
**12**	39.4, C	-	-	35.8, C	-	-
**13**	53.3, CH	2.22	4.0; 12.0; 12.1	51.6, CH	2.35	m
**14**	31.6, CH_2_	2.31 (β); 2.44 (α)	12.1; 17.9; 4.0; 17.9	37.8, CH_2_	1.61 (β); 1.87 (α)	m 3.0; 13.4
**15**	160.8, C	-	-	73.7, C	-	-
**16**	125.3, CH	5.90	s	54.4, CH_2_	2.46; 2.54	13.8; 13.8
**17**	198.3, C	-	-	207.0, C	-	-
**18**	46.2, CH	2.70	5.5;11.3;12.0	50.4, CH	2.73	m
**19**	42.3, CH_2_	2.51 (β); 2.66 (α)	11.3; 13.7; 5.5; 13.7	42.3, CH_2_	2.34 (β); 2.73 (α)	m
**20**	208.7, C	-	-	209.6, C	-	-
**21**	58.5, CH	2.83	s	59.4, CH	2.91	s
**22**	36.1, C	-	-	36.4, C	-	-
**23**	36.1, CH_2_	2.43 (β); 3.70 (α)	20.6; 20.6	36.0, CH_2_	2.36; 3.62	20.4; 20.4
**24**	169.9, C	-	-	172.3, C	-	-
**25**	20.8, CH_3_	1.04	s	18.4, CH_3_	0.99	s
**27**	24.3, CH_3_	2.10	s	31.4, CH_3_	1.45	s
**28**	18.0, CH_3_	0.97	s	18.0, CH_3_	1.00	s
**29**	17.0, CH_3_	1.22	s	18.4, CH_3_	1.17	s
**30**	21.3, CH_3_	0.99	6.4	21.9, CH_3_	0.91	6.6

Norzoanthamide B (**2**) was isolated as an optically active powder, [α]^25^_D_ +10 (*c* 0.03, CHCl_3_), and its molecular formula was established as C_29_H_37_NO_6_ on the basis of the result obtained from ESI-HRMS (*m*/*z* 496.26918; calculated for 496.26936, C_29_H_38_NO_6_, [M + H]^+^). MS data were further supported by analysis of NMR data. Thus, five CH_3_, eight CH_2_ and six CH, as well as ten quaternary carbons (including two carboxylic, two carbonyl and two olefinic carbons) were identified (Table 2). Comparative analysis of the NMR data of **2** with those of norzoanthamine allowed us to conclude that C-1 is now the carboxylic signal at δ_C_ 174.5. Further analyses of COSY, HSQC and HMBC spectra confirmed our previous conclusion. Consequently, an azabicycle [] system was built starting from methine CH-2 (δ_H_ 4.25; δ_C_ 77.0) and sequentially to CH_2_-3 (δ_H_ 1.41/1.86; δ_C_ 32.7), CH-4 (δ_H_ 2.22; δ_C_ 23.8) and, finally, with both CH_2_-5 (δ_H_ 1.18/2.19; δ_C_ 40.7) and methyl CH_3_-30 (δ_H_ 0.99; δ_C_ 21.3). In addition, the HMBC connectivity of proton H-2 with the signal at δ_C_ 174.5 supported the existence of a lactam within this ring. The relative configuration of norzoanthamide B (**2**) was proposed to be 2R*, 4S*, 6S*, 9S*, 10S*, 12S*, 13R*, 18S*, 21R*, 22S*, 23S* based on the correlations observed in the ROESY experiment and supported by a comparison with the chemical shift data reported for norzoanthamine.

**Figure 4:**
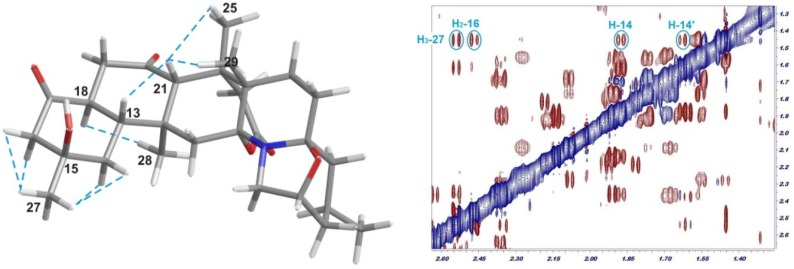
Relevant dipolar correlations observed for 15-hydroxynorzoanthamine (**3**) (dotted blue line) and a selected fragment of the ROESY spectrum.

15-Hydroxynorzoanthamine (**3**) was obtained as a solid, [α]^25^_D_ +8 (*c* 0.03, CHCl_3_). The molecular formula of **3** was deduced by ESI-HRMS as C_29_H_41_NO_6_ (*m*/*z* 500.30050; calculated for 500.30066, [M + H]^+^), therefore indicating the presence of an additional oxygen atom with respect to norzoanthamine. Comparison of the NMR data of **3** with those reported for norzoanthamine clearly revealed that the new metabolite shares the same carbon skeleton, but contains an additional oxygen atom attached to C-15 (Table 2). This is in accordance with the absence of the characteristic olefin carbons C-15 and C-16, as well as with the presence of two new signals at δ_C_ 73.6 and δ_C_ 54.4 corresponding to a tertiary hydroxyl at C-15 and a new methylene at C-16 (δ_H_ 2.46/2.54, d, *J* = 13.8 Hz), respectively (Table 2). These findings were supported by the absence of the α,β-unsaturated ketone distinctive absorption band in the UV spectrum. Analysis of the ROESY experiment confirmed that the relative configurations of all chiral centers in **3** are equivalent to those observed in norzoanthamine. The relative configuration of the new stereogenic center at C-15 was established as R* on the basis of the cross-correlation peak between CH_3_-27 (δ_H_ 1.45) and both CH_2_-14 (δ_H_ 1.61/1.87) and CH_2_-16 (δ_H_ 2.46/2.54) observed in the ROESY spectrum (Figure 4).

## 3. Experimental Section

### 3.1. General Methods

Optical rotations were measured at room temperature in CHCl_3_ using a sodium lamp. Low and high resolution ESI-MS were recorded on a Micromass LTC Premier XE system mass spectrometer (Waters, Milford, CT, USA). NMR spectra were recorded on a Bruker AVANCE III 600 MHz (Bruker, Rheinstetten, Germany) equipped with a 5-mm TCI inverse detection cryo-probe (Bruker, Rheinstetten, Germany). ^1^H and ^13^C NMR chemical shifts were referenced either to the CDCl_3_ peaks at 300 K. COSY, TOCSY, multiplicity-edited HSQC, HMBC and ROESY experiments were performed using standard pulse sequences. HSQC, TOCSY and ROESY experiments were performed in the phase-sensitive mode (States-TPPI or Echo-AntiEcho for quadrature detection in F1) and used gradient coherence selection. ^3^*J*_H,H_ values were measured from 1D ^1^H NMR. TOCSY experiment was recorded using DIPSI during the 50 ms of the isotropic mixing period. The ROESY experiment was recorded using a spin-lock generated by two 180° hard pulses during 400 ms in order to avoid TOCSY artefacts. The HMBC was optimized to detect long-range correlations using a value of 6 Hz. Prior to Fourier transformation, zero filling was performed to expand the data to at least double the number of acquired data points. HPLC separations were carried out with a preparative silica column (10 μ, 19 × 150 mm) (Waters, Milford, CT, USA) and a photodiode array detector (Waters, Milford, CT, USA). TLC plates were visualized by spraying with Dragendorff reagent and phosphomolybdic acid (10% in EtOH), followed by heating.

### 3.2. Biological Material

Colonies of *Zoanthus* sp. were collected by hand in the intertidal zone of Puntal del Hidalgo, Tenerife, Canary Islands (28°34′35.06ʺ N; 16°19′43.64ʺ W). A specimen was deposited at the Department of Animal Biology of University of La Laguna, Tenerife, and classified by Alberto Brito at the University of La Laguna (La Laguna, Tenerife).

### 3.3. Extraction and Chromatographic Separation

Fresh specimens (120 g) were extracted with MeOH (three times using 2 L each time) at room temperature and the solvent removed *in vacuo* to give a brownish viscous oil (1.9 g). The extract was chromatographed on a Sephadex LH-20 column (3 cm of internal diameter and 20 cm of length) (GE Healthcare, Buckinghamshire, UK) using MeOH, collecting 60 tubes of 10 mL, and subsequently with Lobar LiChroprep-RP18 (Merck, Darmstadt, Germany) with MeOH:H_2_O (7:3) as the eluent at 2 mL/min flux. The fractions collected between 8 and 16 min were polled together and further purified by HPLC using a μ-Bondapack C-18 column (Waters, Milford, CT, USA), employing CH_3_CN:MeOH:H_2_O (2:1:1) as the mobile phase at 2 mL/min to obtain norzoanthamide B (**2**) (0.45 mg; 15–17 min). In addition, fractions polled from the Lobar LiChroprep-RP18 chromatographic step (Merck, Darmstadt, Germany) between 17 and 25 min were further purified by HPLC using a μ-Bondapack C-18 column, employing CH_3_CN:MeOH:H_2_O (2:1:2) as the eluent at 2 mL/min to yield 2-hydroxi-11-ketonorzoanthamide B (**1**) (0.5 mg; 15–18 min) and 15-hydroxynorzoanthamine (**3**) (0.3 mg; 22–24 min).

2-Hydroxy-11-ketonorzoanthamide B (**1**): white powder; [α]^25^_D_ +9 (*c* 0.045, CHCl_3_); UV λ_max_ (CHCl_3_) 239.8 (ε 1,630) (log ε = 3.2); IRνmax (CHCl_3_) 3384, 2927, 2857, 1742, 1723, 1669, 1383, 1220 and 1008 cm^−1^; ESI-HRMS *m*/*z* 526.24340 (calculated for C_29_H_36_NO_8_, 526.24354, [M + H]^+^); ^1^H (600 MHz, CDCl_3_) and ^13^C (150 MHz, CDCl_3_) NMR chemical shifts are reported in Table 1.

Norzoanthamide B (**2**): white powder; [α]^25^_D_ +10 (*c* 0.03, CHCl_3_); UV λ_max_ (CHCl_3_) 239.8 (ε 1630) (log ε = 3.2); IRνmax (CHCl_3_) 3455, 2926, 2856, 1726, 1465, 1383 and 1246 cm^−1^; ESI-HRMS *m*/*z* 496.26918 (calculated For C_29_H_38_NO_6_, 496.26936, [M + H]^+^); ^1^H (600 MHz, CDCl_3_) and ^13^C (150 MHz, CDCl_3_) NMR chemical shifts are reported in Table 2.

15-Hydroxynorzoanthamine (**3**): white powder; [α]^25^_D_ +8 (*c* 0.03, CHCl_3_); IRνmax (CHCl_3_) 3414, 2924, 2854, 1715, 1665, 1377, 1240 and 1217 cm^−1^; ESI-HRMS *m*/*z* 500.30050 (calculated for C_29_H_42_O_6_N, 500.30066, [M + H]^+^); ^1^H (600 MHz, CDCl_3_) and ^13^C (150 MHz, CDCl_3_) NMR chemical shifts are reported in Table 2.

## 4. Conclusions

The structures of three new congeners of norzoanthamine have been elucidated by means of MS and NMR spectroscopy. Despite, from our point of view, the new metabolites described here not providing new insights into the intriguing biogenetic origin of this class of molecules, their isolation does provide additional evidence of the currently accepted hypothesis regarding the biosynthesis of this family of natural products.

## Supplementary Files

Supplementary File 1

## Supplementary Information

Experimental procedures and spectral data for each compound.
