# Supplementary material for: New Oxidized Zoanthamines from a Canary Islands Zoanthus sp
Source: Mar Drugs. 2014 Oct 14;12(10):5188–96. doi: 10.3390/md12105188 (PMC4210893; doi:10.3390/md12105188)
Supplement: Supplementary File 1 [file marinedrugs-12-05188-s001.pdf]

Supplementary Information

Table of Contents

Isolation procedure for 2-hydroxy-11-ketonorzoanthamide B (**1**), norzoanthamide B (**2**) and 15-hydroxynorzoanthamine (**3**)	**S2**
NMR chemical shifts of 2-hydroxy-1-ketonorzoanthamide B (**1**)	**S3**
^{1}H-NMR spectrum of 2-hydroxy-11-ketonorzoanthamide B (**1**)	**S4**
^{13}C-NMR spectrum of 2-hydroxy-11-ketonorzoanthamide B (**1**)	**S5**
COSY spectrum of 2-hydroxy-11-ketonorzoanthamide B (**1**)	**S6**
HSQC spectrum of 2-hydroxy-11-ketonorzoanthamide B (**1**)	**S7**
HMBC spectrum of 2-hydroxy-11-ketonorzoanthamide B (**1**)	**S8**
ROESY spectrum of 2-hydroxy-11-ketonorzoanthamide B (**1**)	**S9**
MS spectrum of 2-hydroxy-11-ketonorzoanthamide B (**1**)	**S10**
NMR chemical shifts assignments of norzoanthamide B (**2**)	**S11**
^{1}H-NMR spectrum of norzoanthamide B (**2**)	**S12**
^{13}C-NMR spectrum of norzoanthamide B (**2**)	**S13**
COSY spectrum of norzoanthamide B (**2**)	**S14**
HSQC spectrum of norzoanthamide B (**2**)	**S15**
HMBC spectrum of norzoanthamide B (**2**)	**S16**
ROESY spectrum of norzoanthamide B (**2**)	**S17**
MS spectrum of norzoanthamide B (**2**)	**S18**
NMR chemical shifts of 15-hydroxynorzoanthamine (**3**)	**S19**
^{1}H-NMR spectrum of 15-hydroxynorzoanthamine (**3**)	**S20**
^{13}C-NMR spectrum of 15-hydroxynorzoanthamine (**3**)	**S21**
COSY spectrum of 15-hydroxynorzoanthamine (**3**)	**S22**
HSQC spectrum of 15-hydroxynorzoanthamine (**3**)	**S23**
HMBC spectrum of 15-hydroxynorzoanthamine (**3**)	**S24**
ROESY spectrum of 15-hydroxynorzoanthamine (**3**)	**S25**
MS spectrum of 15-hydroxynorzoanthamine (**3**)	**S26**

Figure S1. Isolation procedure for 2-hydroxy-11-ketonorzoanthamide B (**1**), norzoanthamide B (**2**) and 15-hydroxynorzoanthamine (**3**).

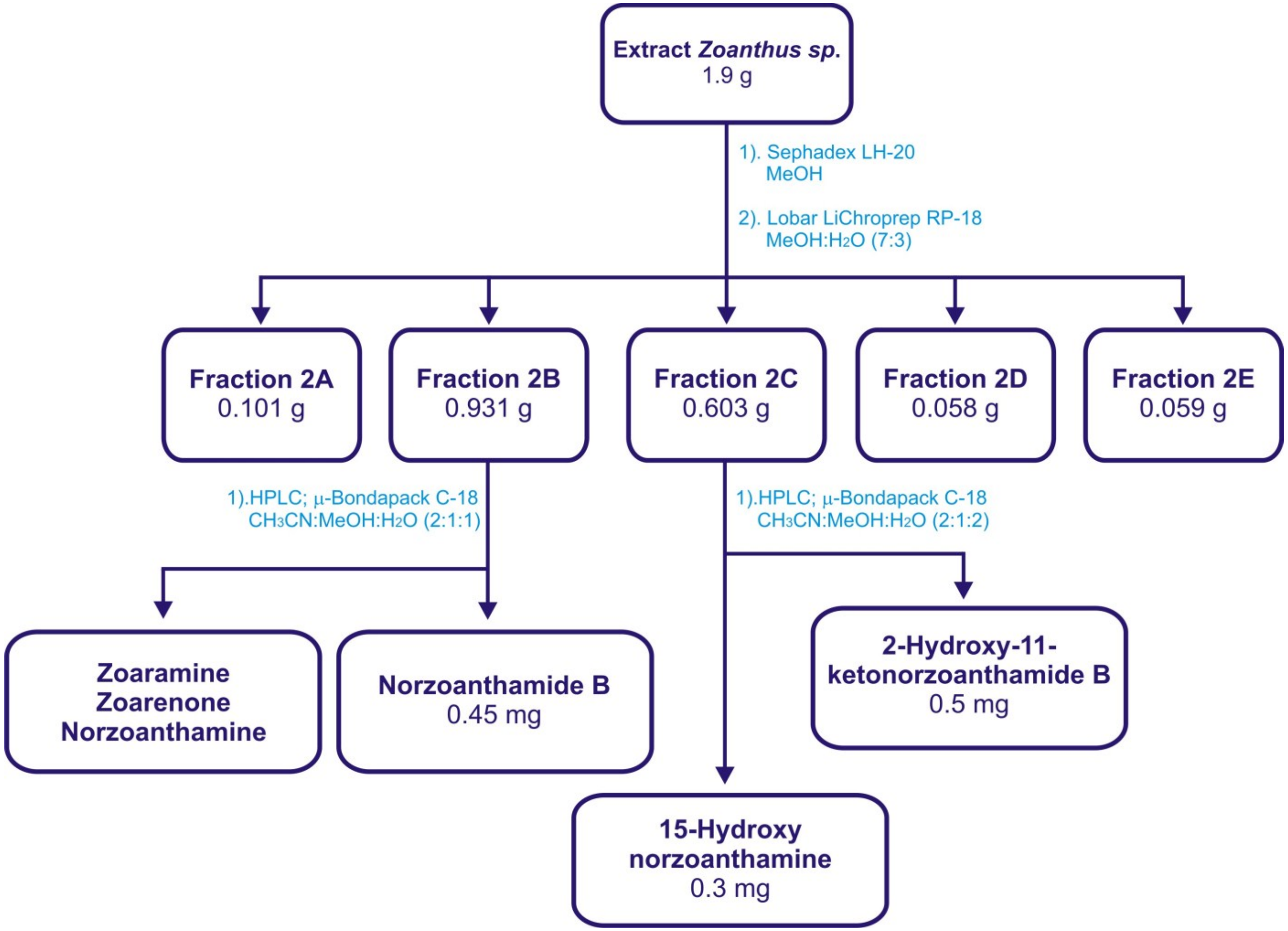

Table S1. NMR chemical shifts of 2-hydroxy-11-ketonorzoanthamide B (**1**).

***n* °C**	**δ ^{13}C**	**δ ^{1}H**	**Multiplicity**	***J* (Hz)**
**1**	170.6			
**2**	100.6			
**3**	38.0	1.43; 2.03	dd; dd	11.2; 12.0
**4**	24.5	2.05	m	
**5**	40.3	1.15; 2.05	dd; dd	12.3; 14.8
**6**	90.2			
**7**	28.7	1.92; 2.14		2.9; 4.0; 13.7; 3.1; 12.1; 13.7
**8**	23.8	1.70; 1.86		3.1; 4.0; 13.4; 2.9; 12.1; 13.4
**9**	43.9			
**10**	95.7			
**11**	195.8			
**12**	54.9			
**13**	46.7	2.76	dd	3.6; 10.3; 12.0
**14**	32.7	2.17; 2.98	dd; dd	10.3; 18.2; 3.6; 18.2
**15**	162.2			
**16**	124.5	5.90	s	
**17**	197.9			
**18**	46.0	2.70	dd	5.8; 11.3; 12.0
**19**	42.7	2.48; 2.67	dd; dd	11.3; 13.3; 5.8; 13.3
**20**	206.9			
**21**	60.8	2.89	s	
**22**	38.1			
**23**	34.6	2.55; 4.27	d; d	20.6; 20.6
**24**	168.1			
**25**	20.9	1.01	s	
**27**	24.4	2.01	s	
**28**	14.1	1.34	s	
**29**	17.7	1.03	s	
**30**	20.9	1.00	d	6.0

Figure S2. ^{1}H-NMR spectrum of 2-hydroxy-11-ketonorzoanthamide B (**1**).

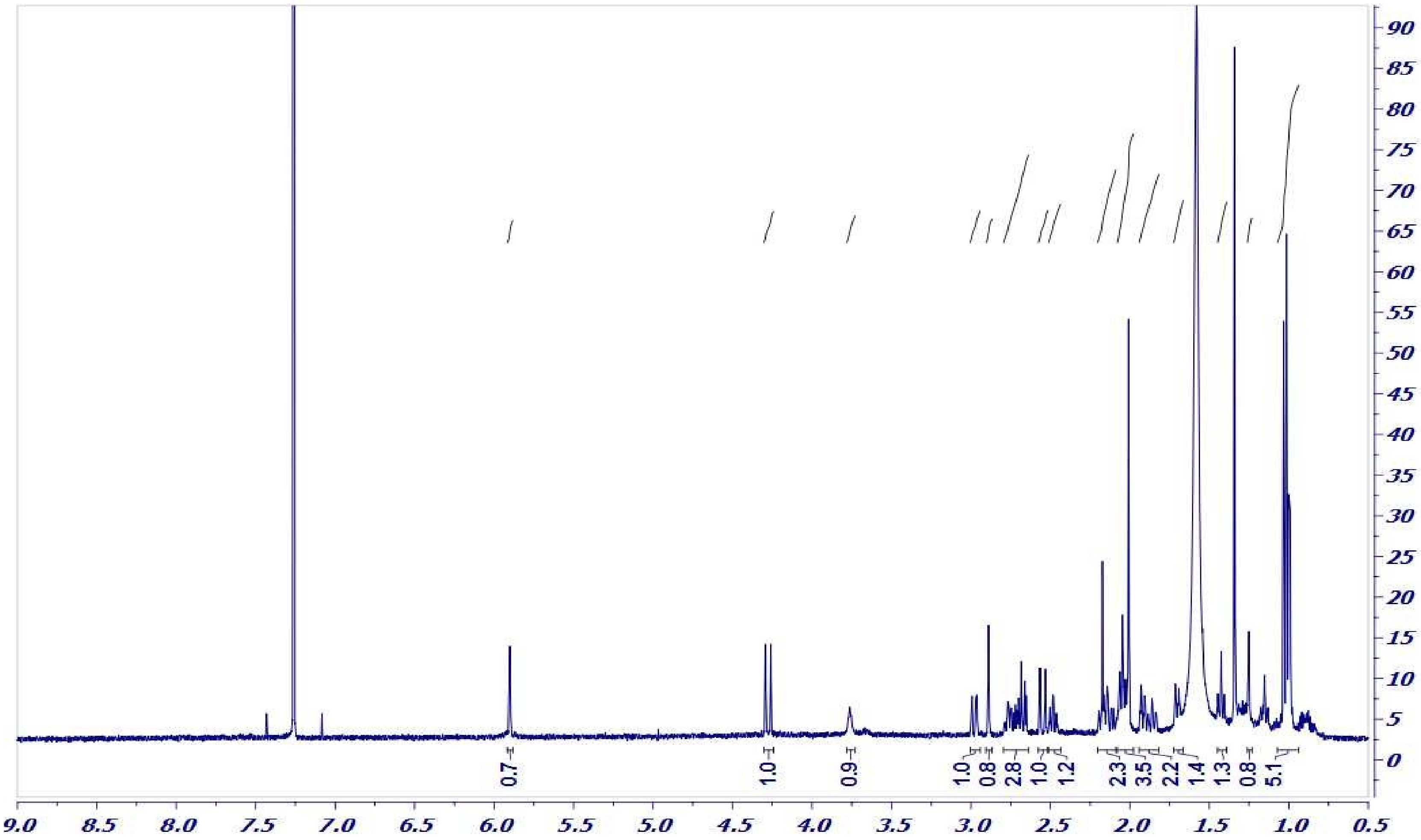

Figure S3. ^{13}C-NMR spectrum of 2-hydroxy-11-ketonorzoanthamide B (**1**).

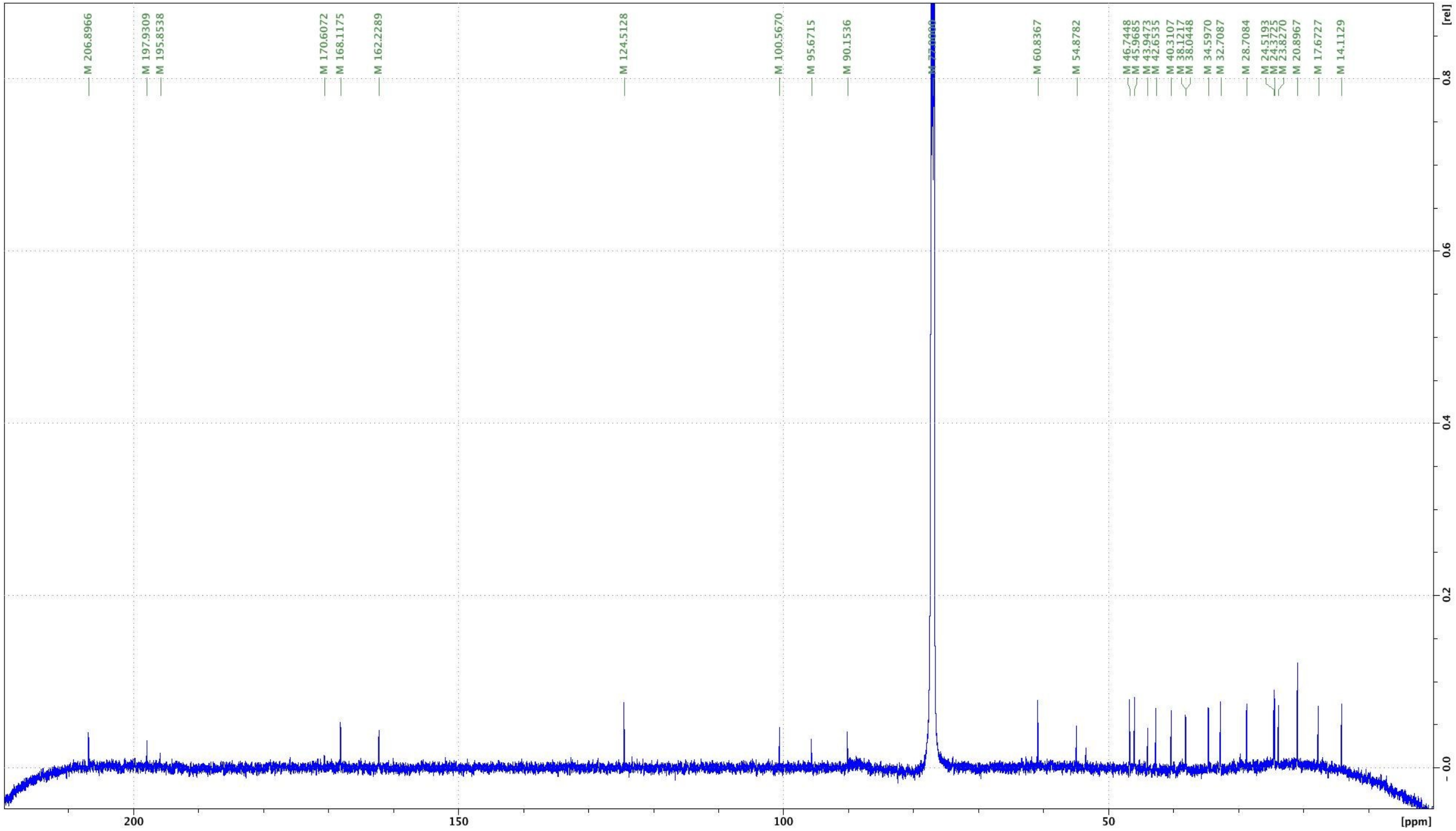

Figure S4. COSY spectrum of 2-hydroxy-11-ketonorzoanthamide B (**1**).

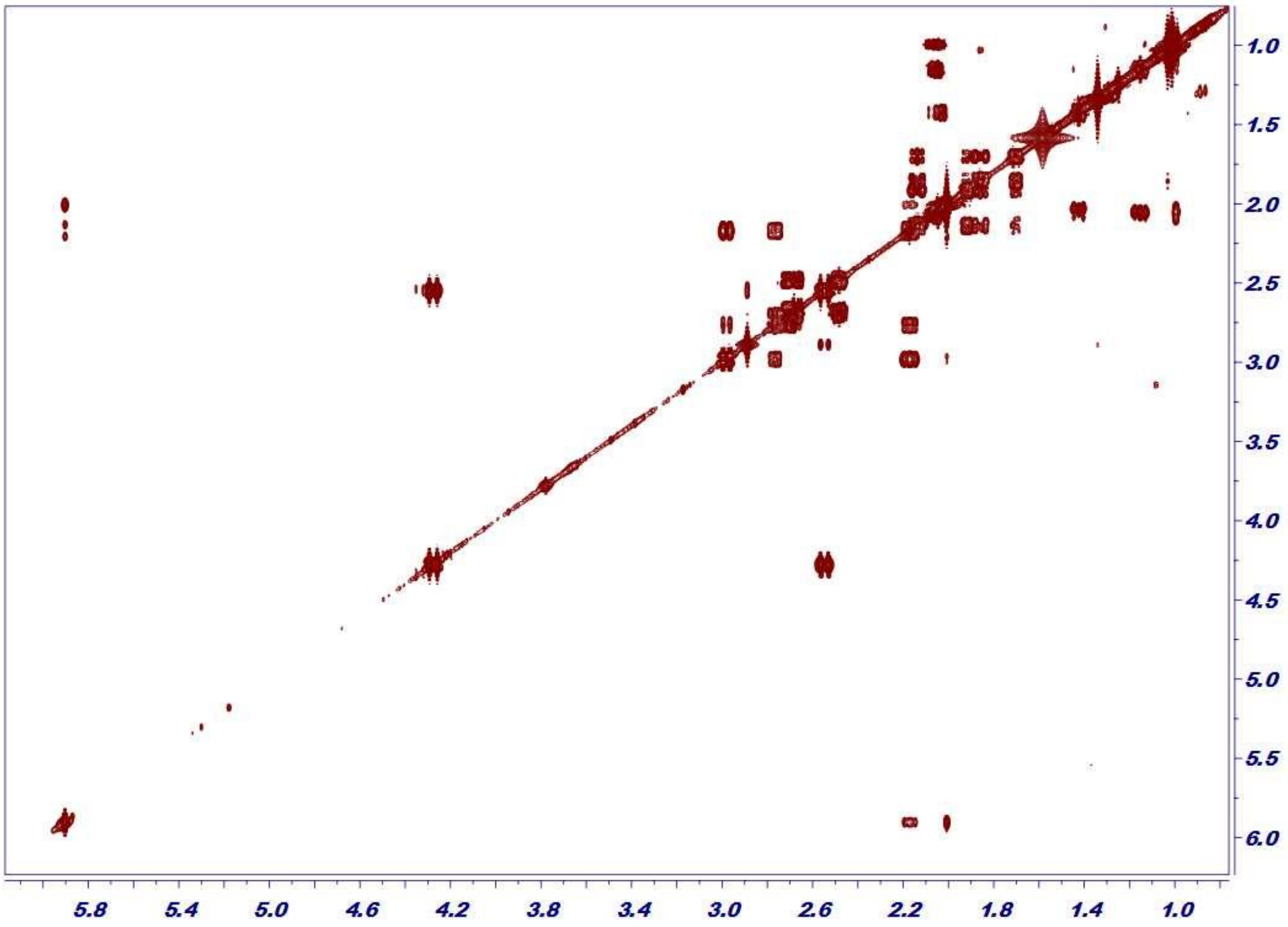

Figure S5. HSQC spectrum of 2-hydroxy-11-ketonorzoanthamide B (**1**).

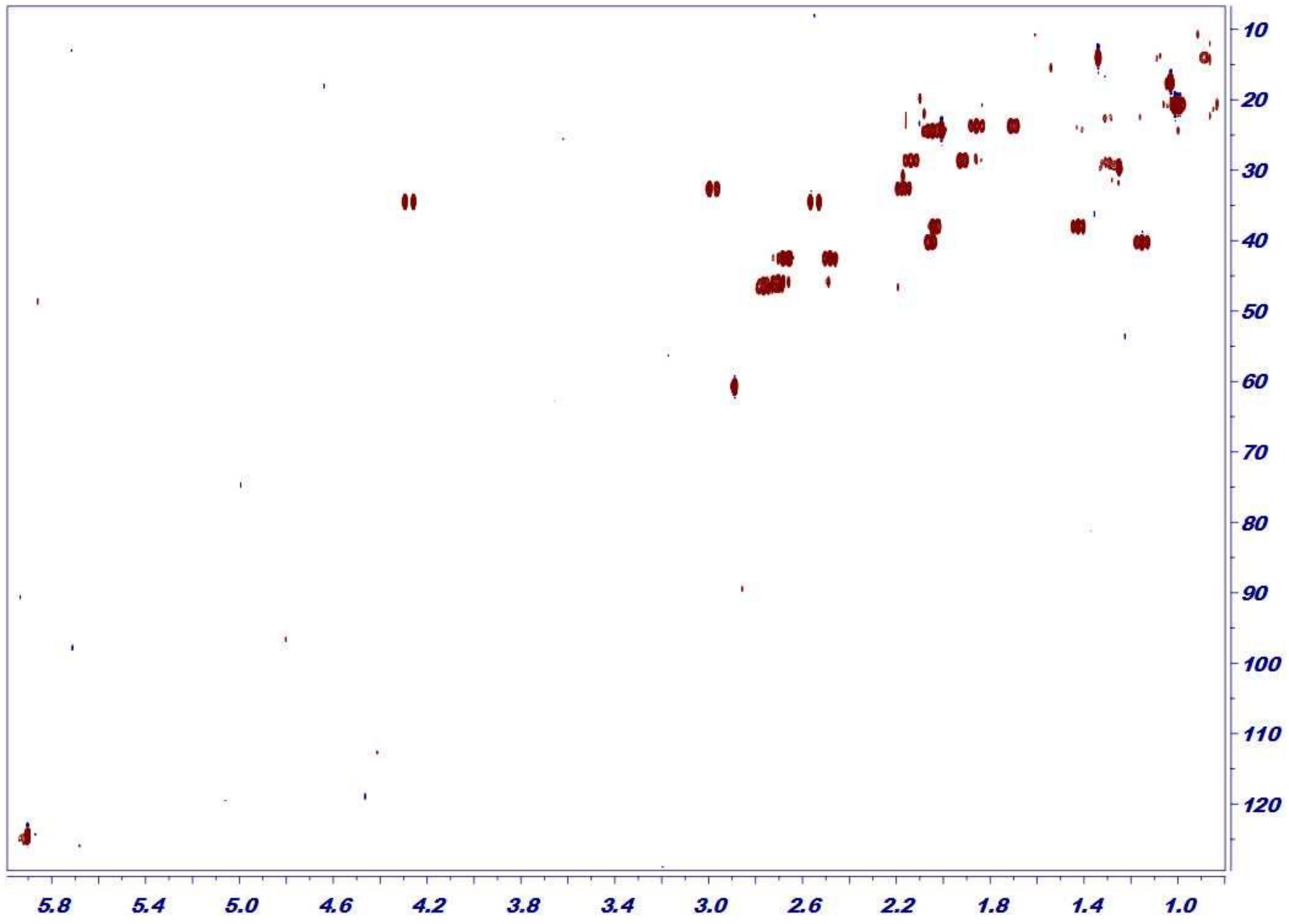

Figure S6. HMBC spectrum of 2-hydroxy-11-ketonorzoanthamide B (**1**).

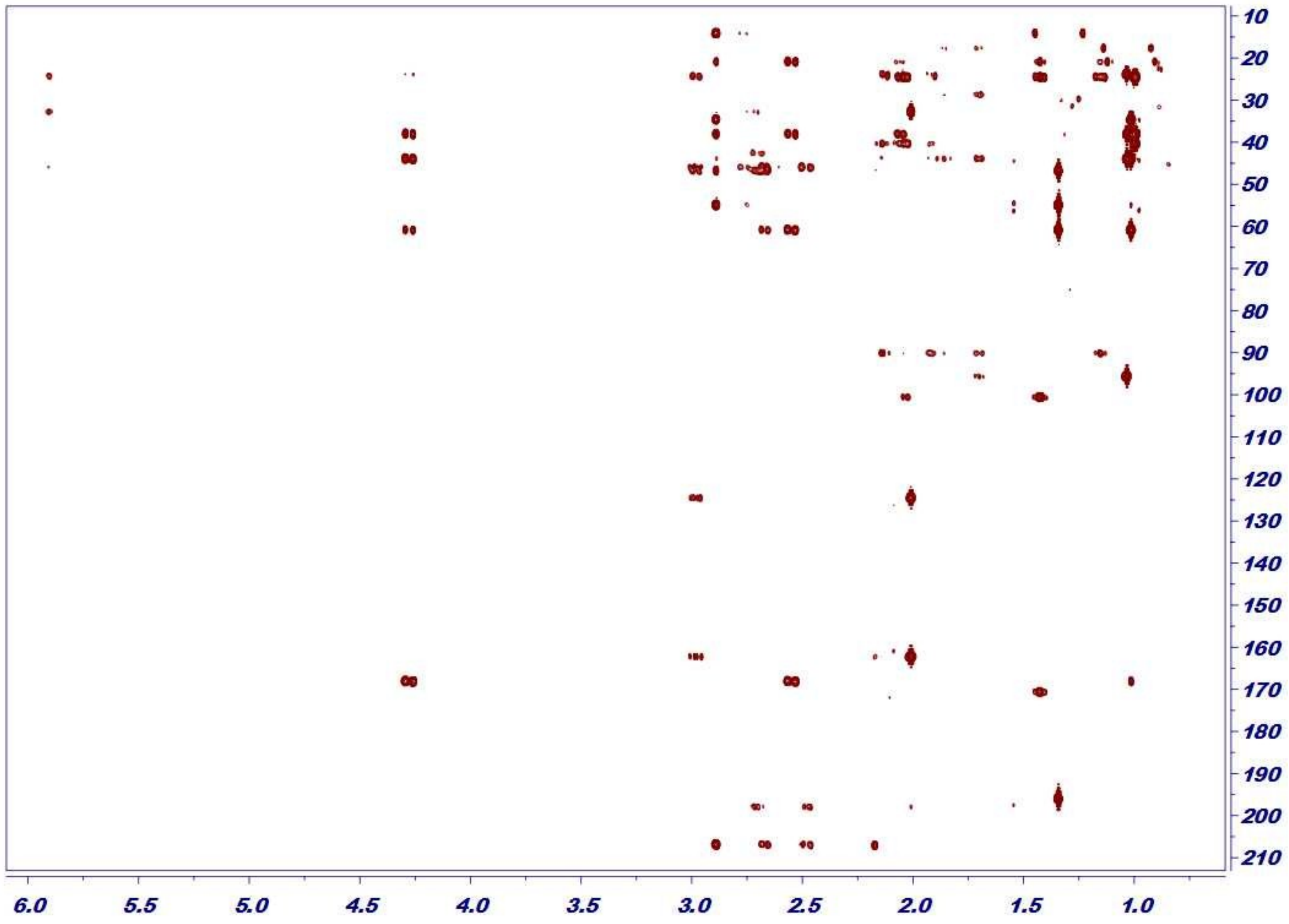

Figure S7. ROESY spectrum of 2-hydroxy-11-ketonorzoanthamide B (**1**).

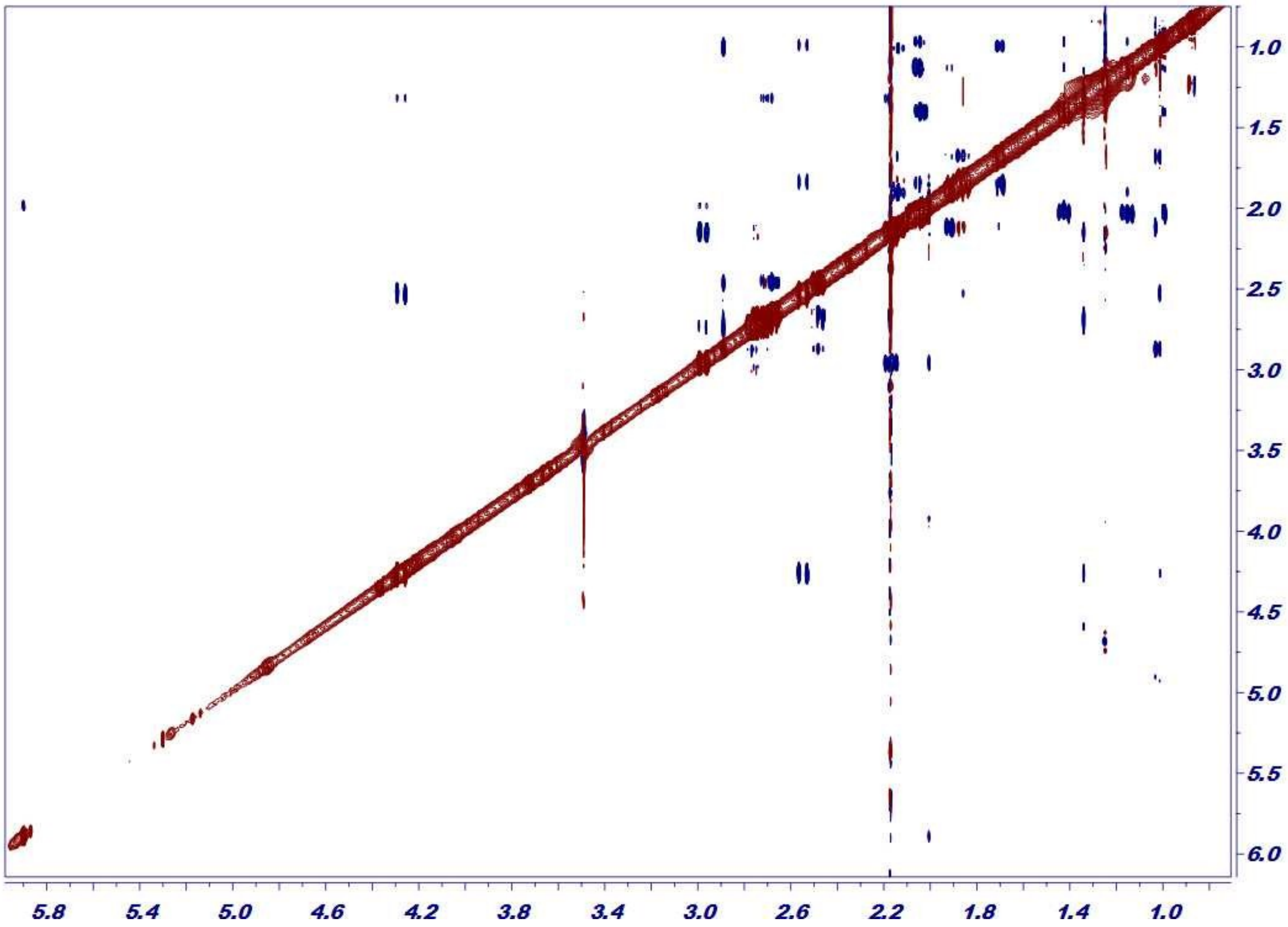

Figure S8. MS spectrum of 2-hydroxy-11-ketonorzoanthamide B (**1**).

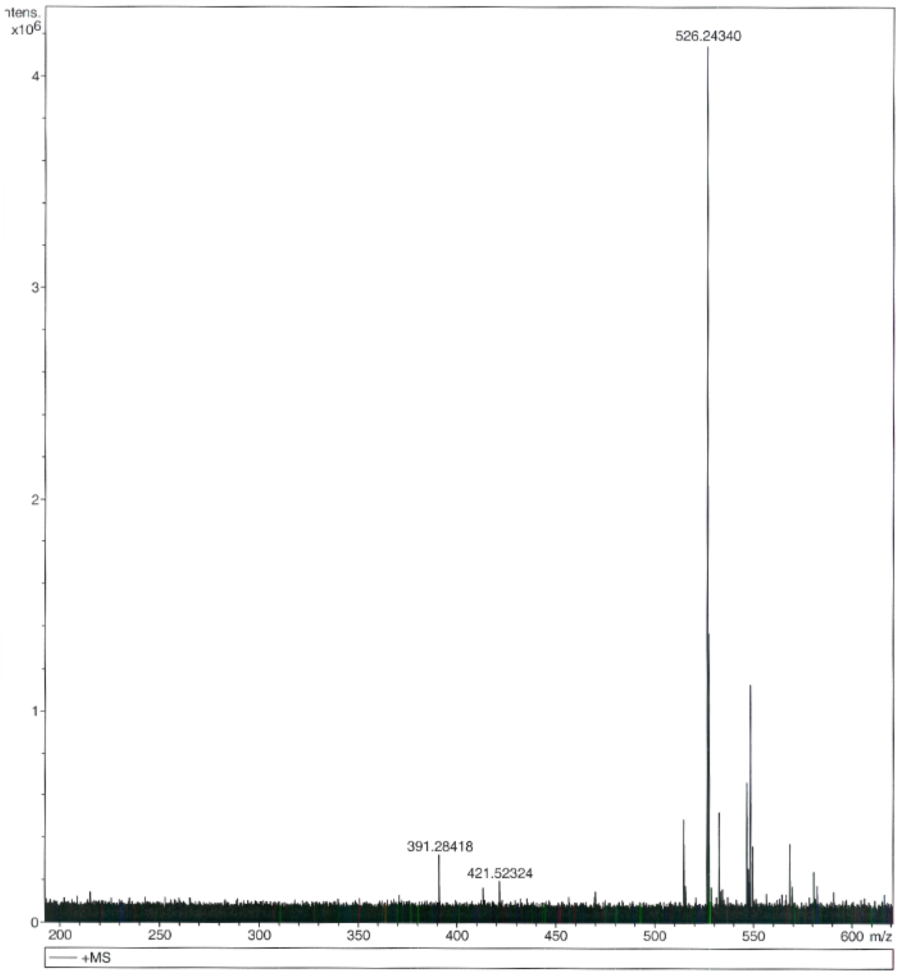

Table S2. NMR chemical shifts of norzoanthamide B (**2**).

n °C	δ ^{13}C	δ^{1}H	Multiplicity	*J* (Hz)
**1**	174.5			
**2**	77.0	4.25	dd	2.1; 3.6
**3**	32.7	1.41; 1.86	ddd; ddd	3.6; 12.0; 13.5; 2.1; 4.5; 13.5
**4**	23.8	2.22	dddd	4.2; 4.5; 11.1; 12.0
**5**	40.7	1.18; 2.19	dd; dd	11.1; 13.2; 4.2; 13.2
**6**	93.4			
**7**	29.7	1.90; 2.08	ddd; ddd	3.1; 4.2; 13.5; 5.6; 13.3; 13.5
**8**	22.6	1.63; 1.65	m; m	
**9**	39.8			
**10**	96.6			
**11**	40.3	2.30; 3.52	d; d	15.2; 15.2
**12**	39.7			
**13**	53.3	2.22	dd	4.0; 12.0; 12.1;
**14**	31.9	2.31; 2.44	dd; dd	12.1; 17.9; 4.0; 17.9
**15**	160.6			
**16**	125.3	5.90	s	
**17**	198.3			
**18**	46.2	2.70	dd	5.5; 11.3; 12.0
**19**	42.3	2.51; 2.66	dd; dd	11.3; 13.7; 5.5; 13.7
**20**	208.7			
**21**	58.5	2.83	s	
**22**	36.1			
**23**	36.1	2.43; 3.70	d; d	20.6; 20.6
**24**	170.0			
**25**	20.8	1.04	s	
**27**	24.2	2.10	s	
**28**	17.9	0.97	s	
**29**	16.9	1.22	s	
**30**	21.3	0.99	d	6.4

Figure S9. ^{1}H-NMR spectrum of norzoanthamide B (**2**).

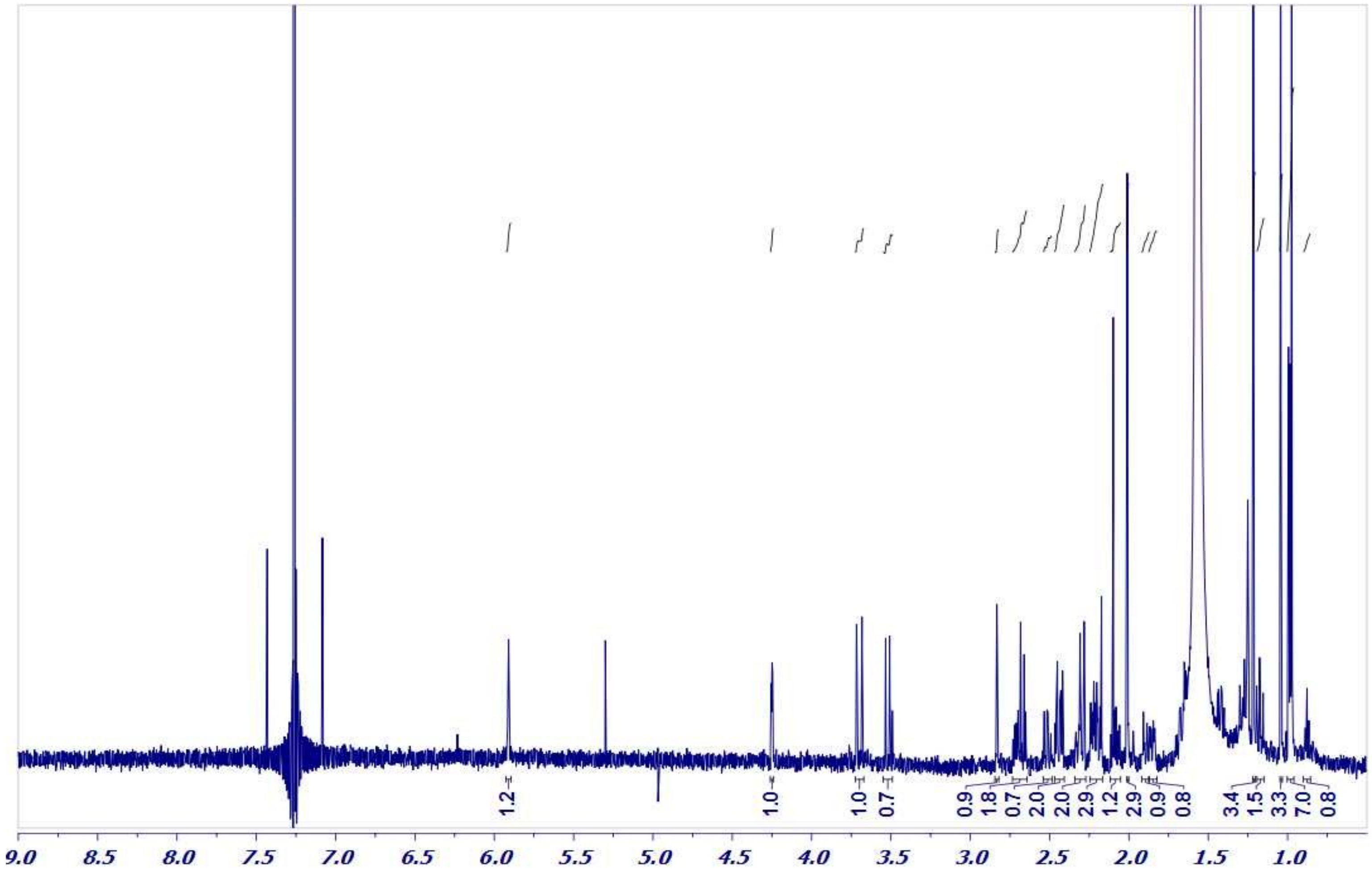

Figure S10. ^{13}C-NMR spectrum of norzoanthamide B (**2**).

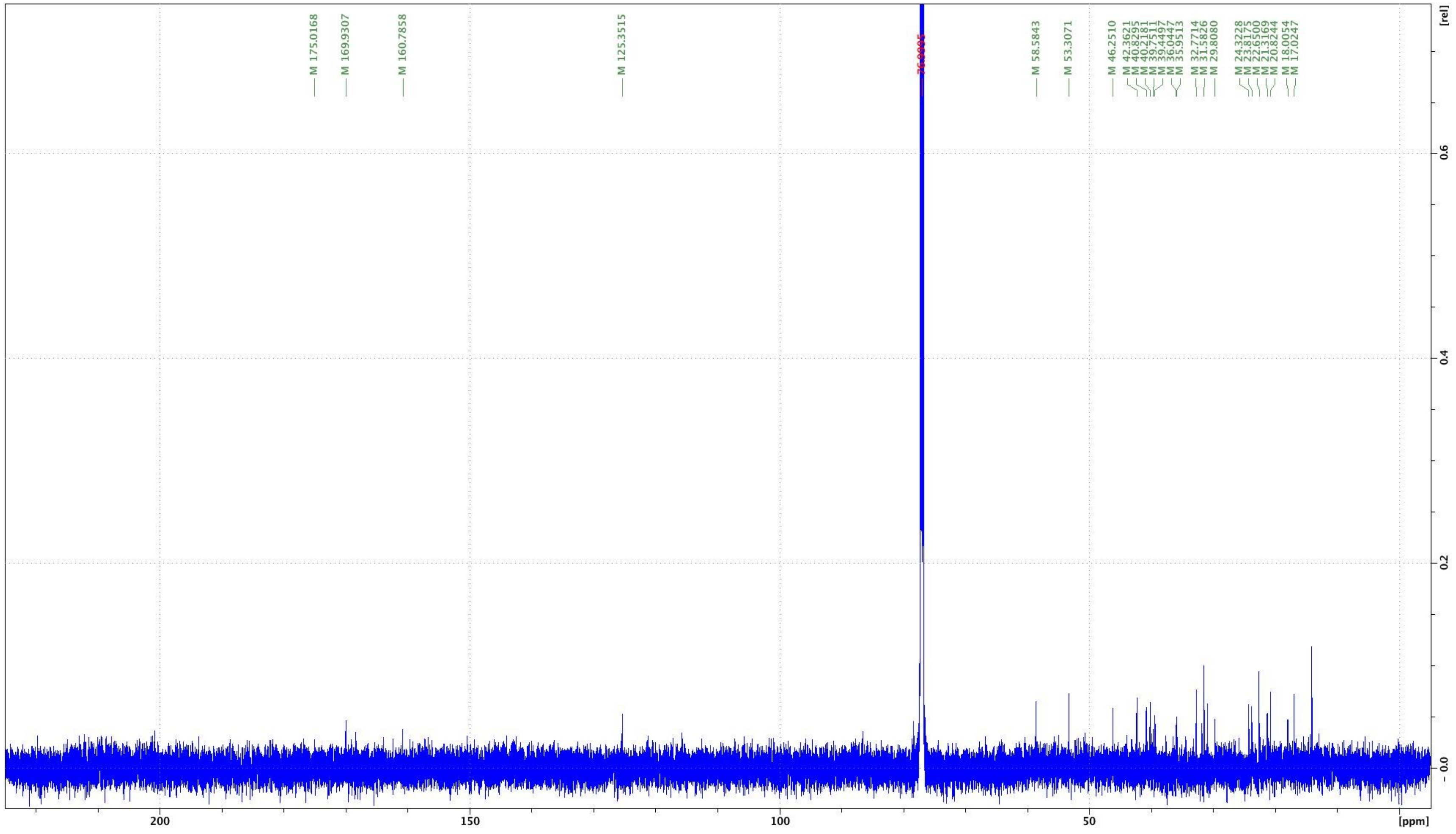

Figure S11. COSY spectrum of norzoanthamide B (**2**).

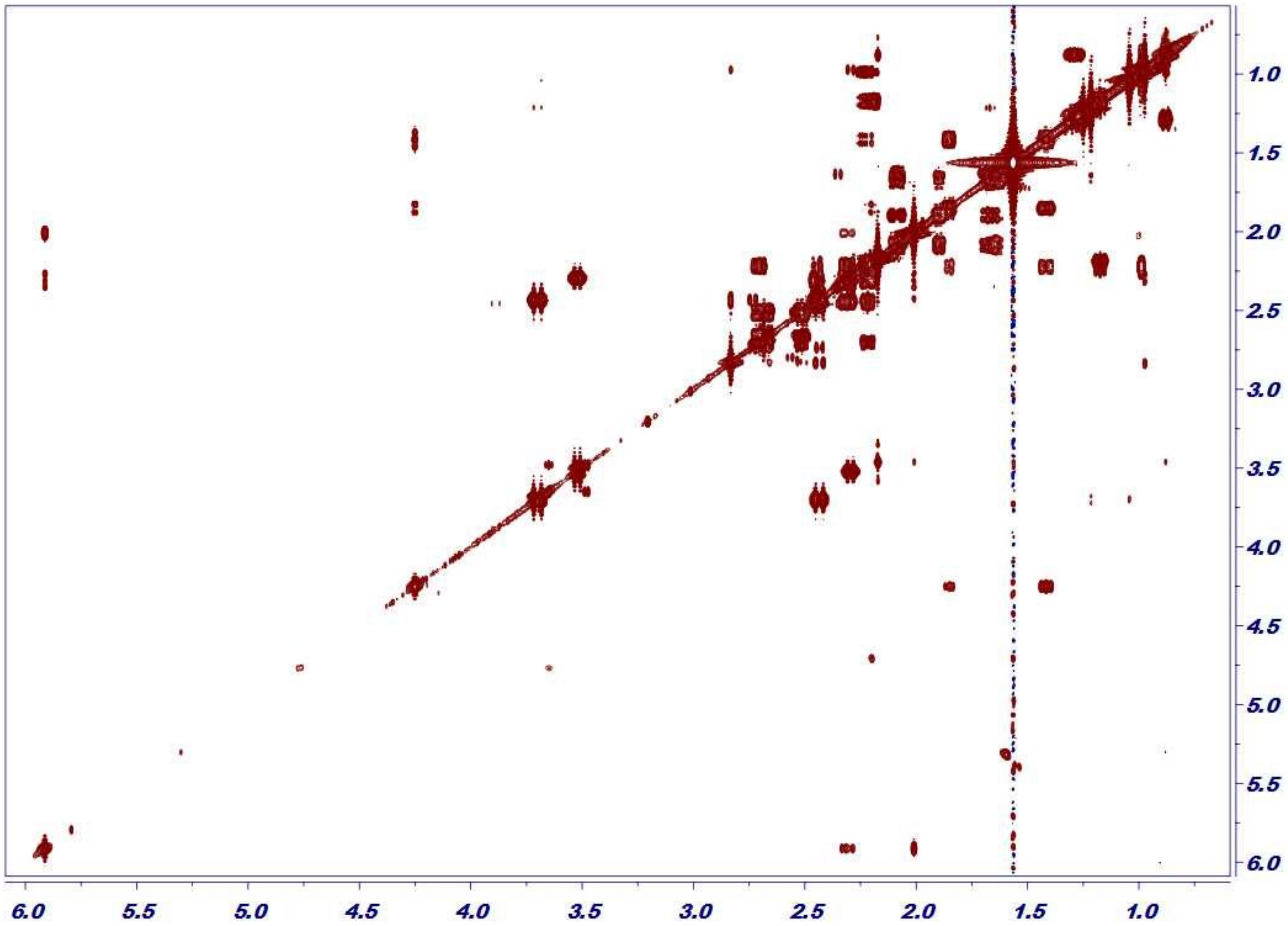

Figure S12. HSQC spectrum of norzoanthamide B (**2**).

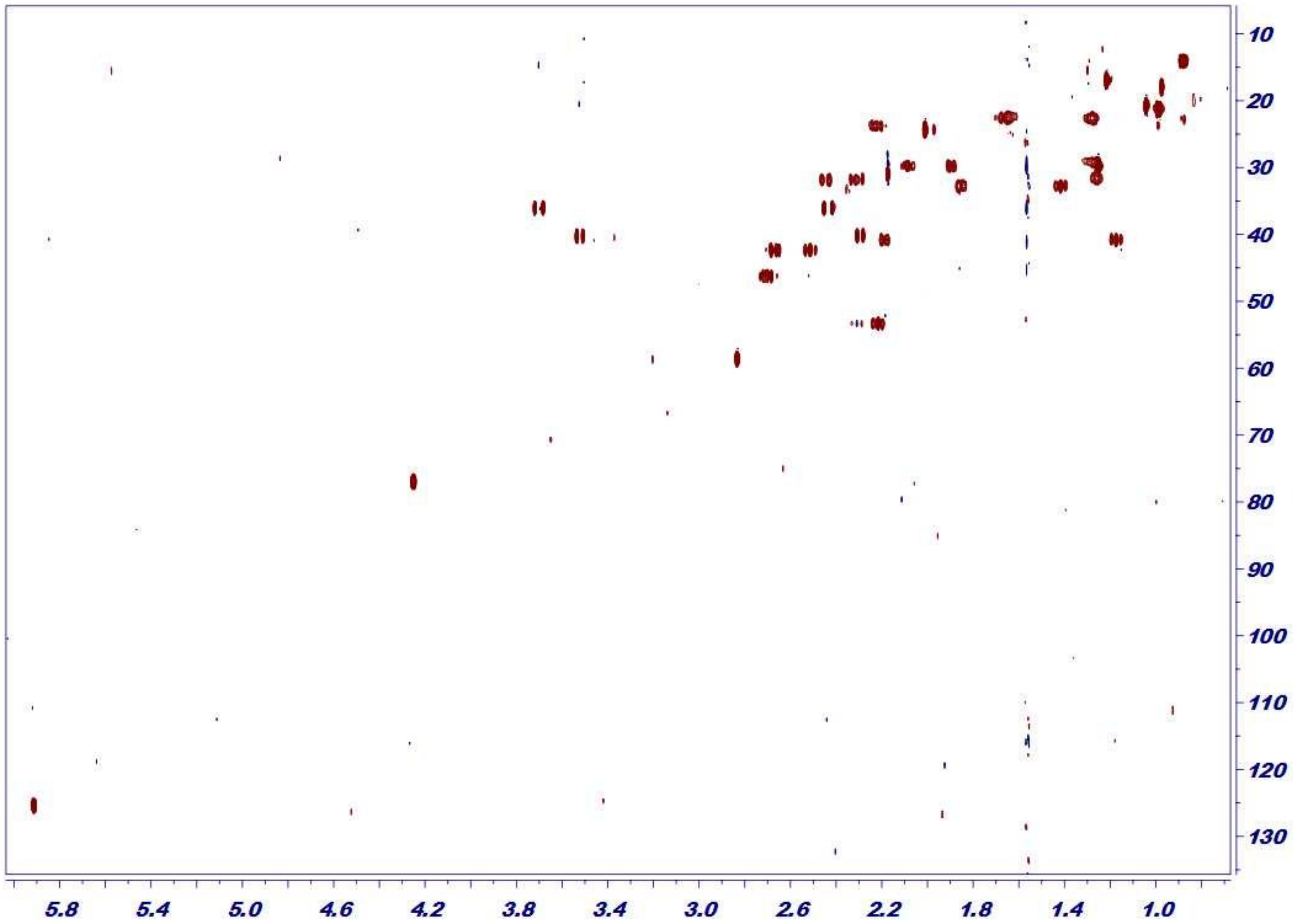

Figure S13. HMBC spectrum of norzoanthamide B (**2**).

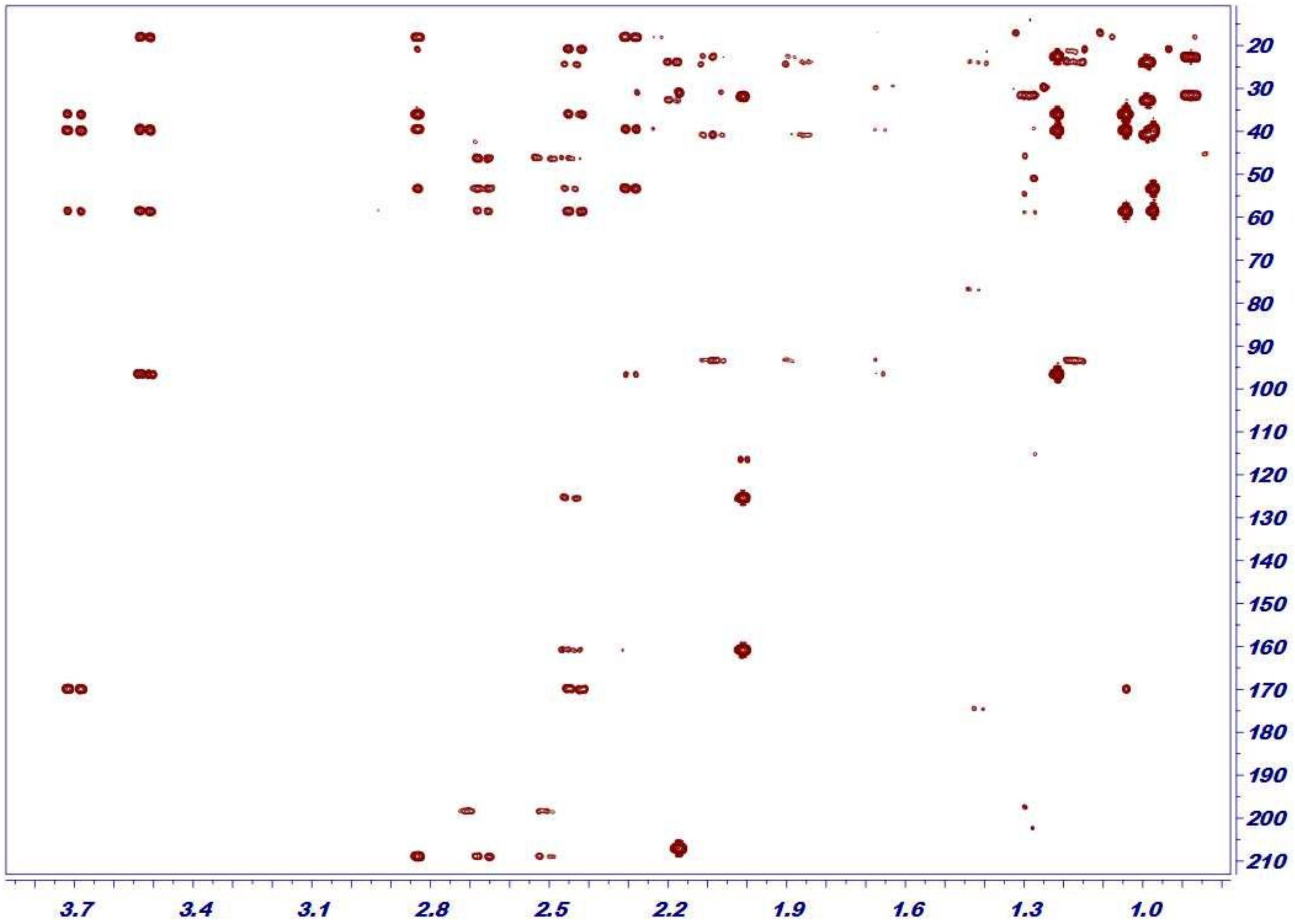

Figure S14. ROESY spectrum of norzoanthamide B (**2**).

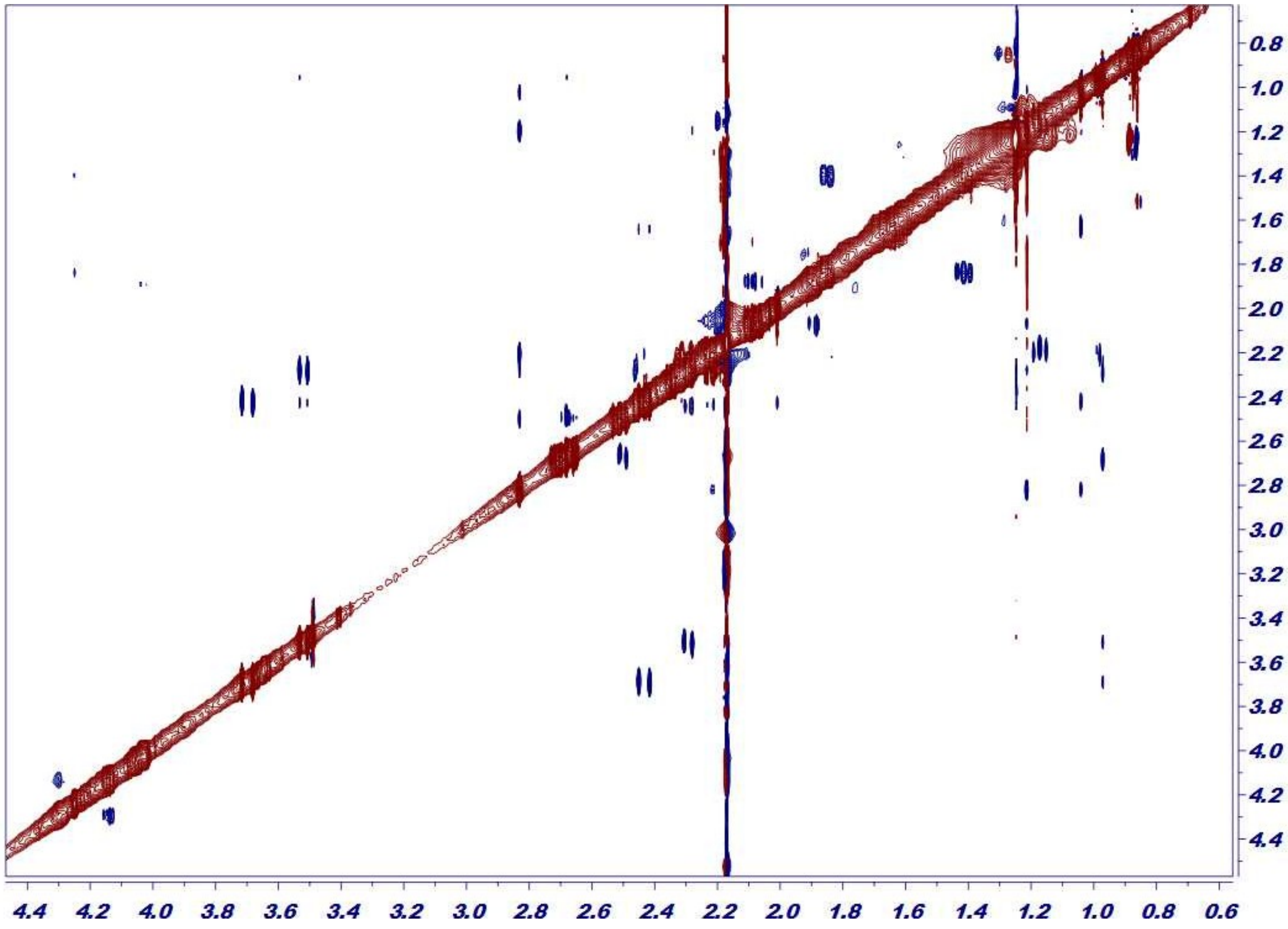

Figure S15. MS spectrum of norzoanthamide B (**2**).

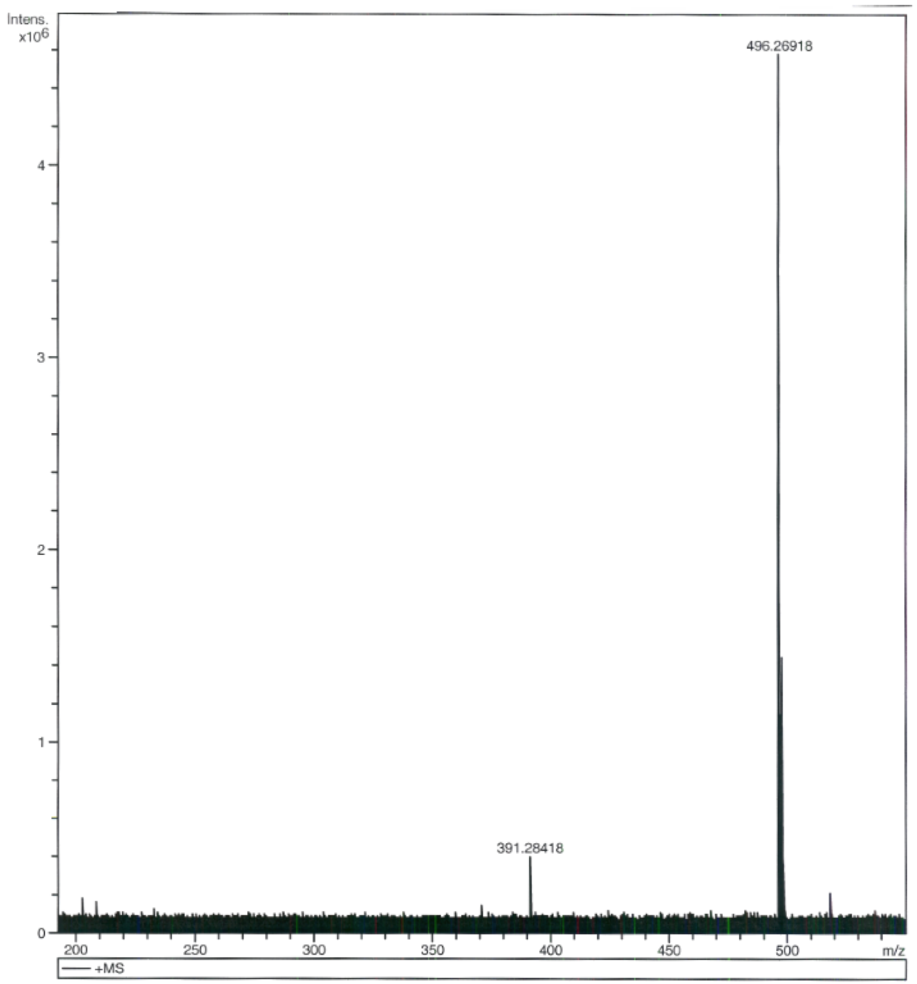

Table S3. NMR chemical shifts of 15-hydroxynorzoanthamine (**3**).

***n* °C**	**δ ^{13}C**	**δ^{1}H**	**Multiplicity**	***J* (Hz)**
**1**	47.1	3.23; 3.25	m; m	
**2**	74.2	4.55	m	
**3**	39.0	1.46; 1.55	m; m	
**4**	22.9	2.27	m	6.0; 11.1
**5**	44.4	1.08; 2.08	dd; dd	11.1; 14.0; 6.0; 14.0
**6**	89.8			
**7**	29.9	1.76; 1.89	m; m	
**8**	23.7	1.55; 1.67	m; m	
**9**	39.9			
**10**	101.9			
**11**	42.3	1.90; 2.13	d; d	14.0; 14.0
**12**	35.6			
**13**	51.6	2.35	m	
**14**	37.8	1.61; 1.87	m; dd	3.0; 13.4
**15**	73.6			
**16**	54.4	2.46; 2.54	d; d	13.8; 13.8
**17**	207.0			
**18**	50.4	2.73	m	
**19**	42.3	2.34; 2.73	m; m	
**20**	209.6			
**21**	59.3	2.91	s	
**22**	36.4			
**23**	36.0	2.36; 3.62	d; d	20.4; 20.4
**24**	172.3			
**25**	18.5	0.99	s	
**27**	31.4	1.45	s	
**28**	18.2	1.00	s	
**29**	18.5	1.17	s	
**30**	21.9	0.91	d	6.6

Figure S16. ^{1}H-NMR spectrum of 15-hydroxynorzoanthamine (**3**).

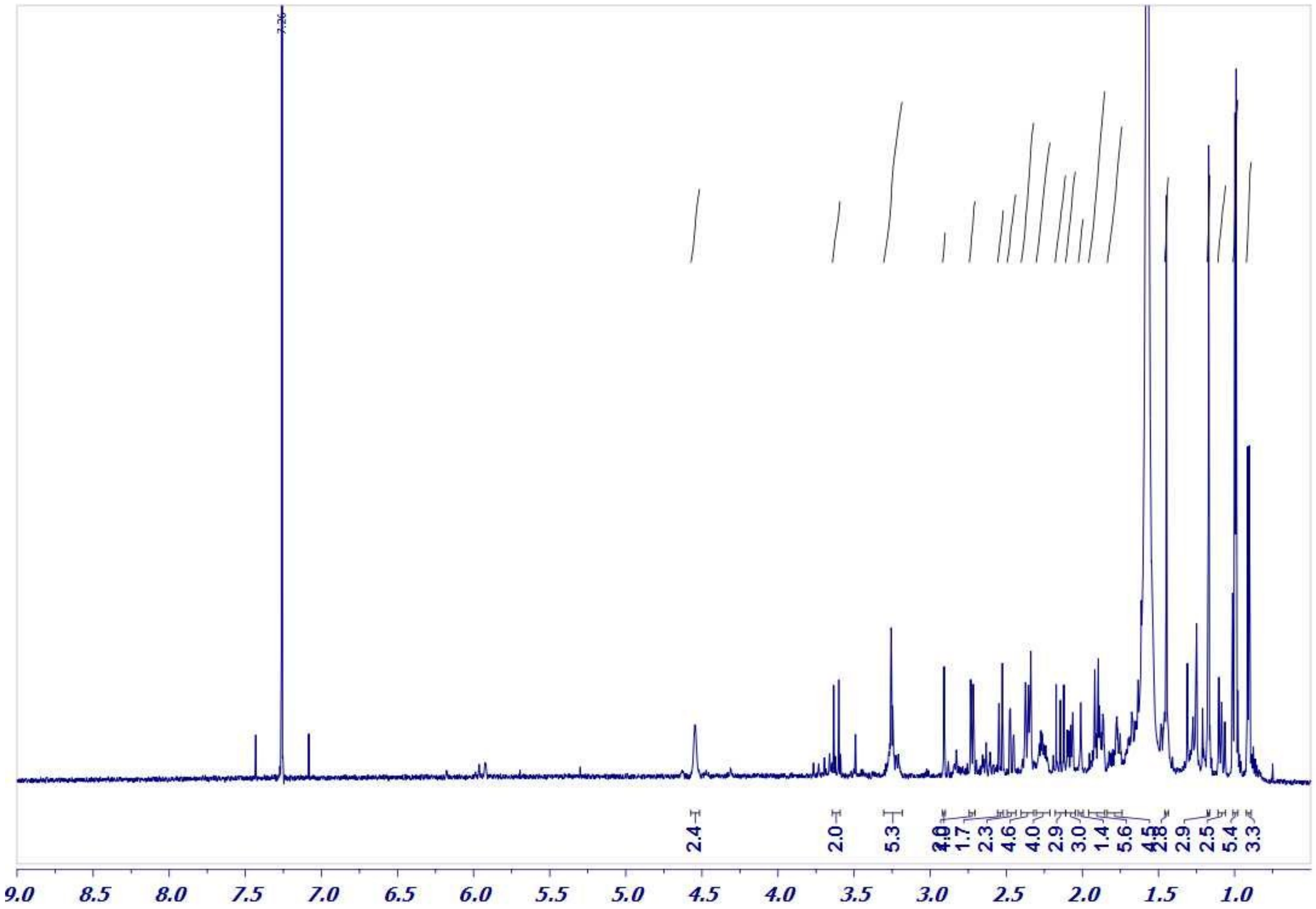

Figure S17. ^{13}C-NMR spectrum of 15-hydroxynorzoanthamine (**3**).

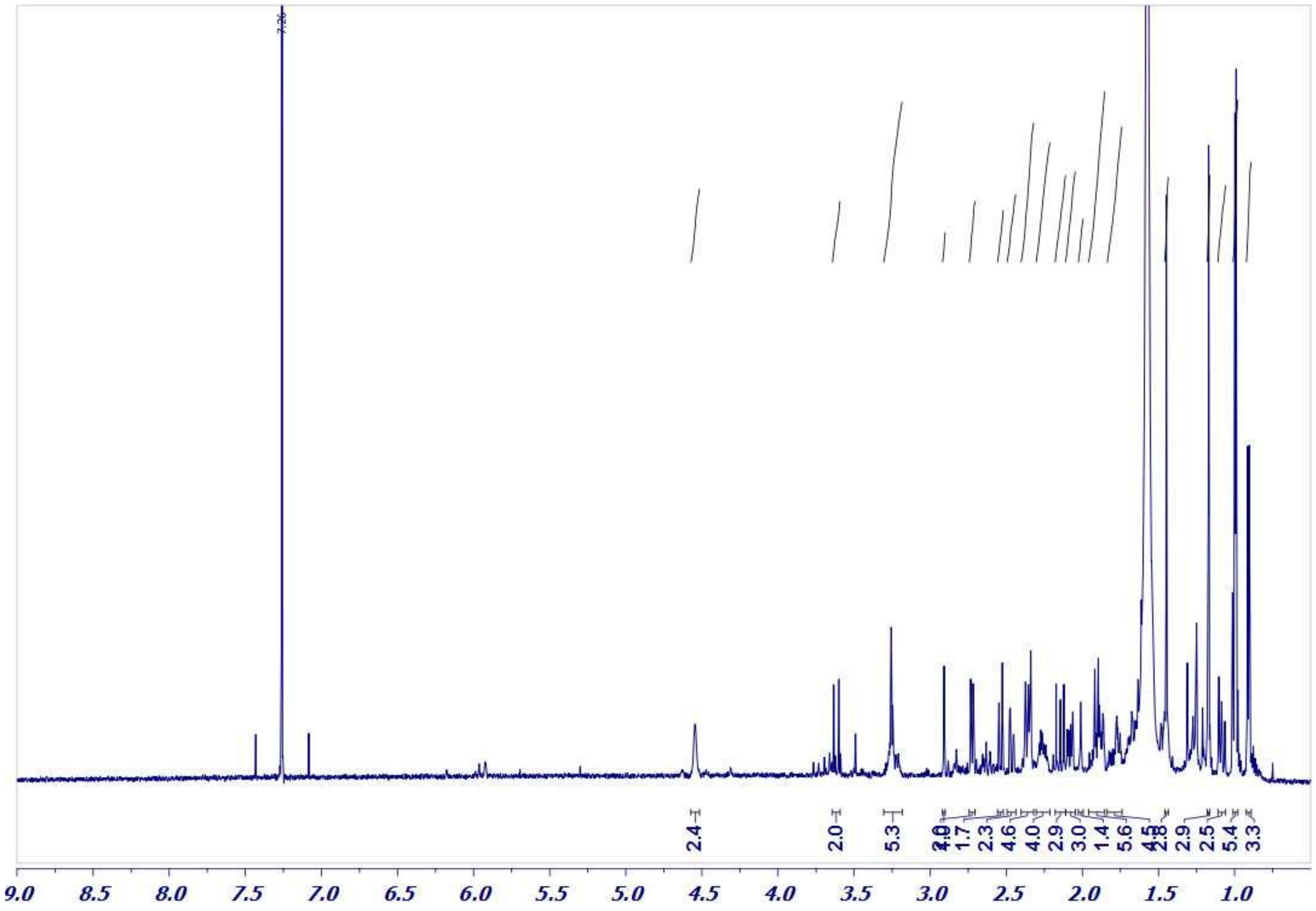

Figure S18. COSY spectrum of 15-hydroxynorzoanthamine (**3**).

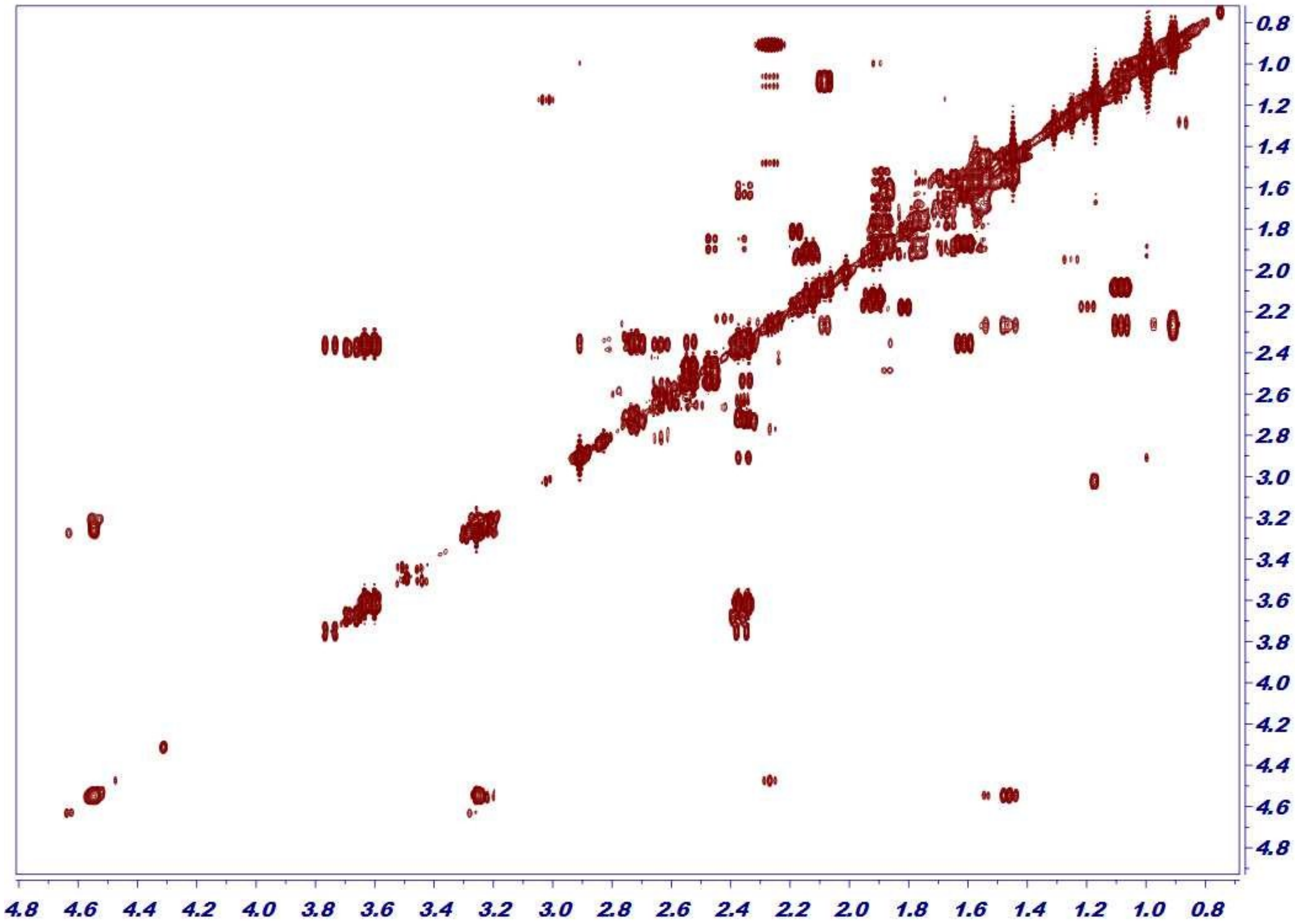

Figure S19. HSQC spectrum of 15-hydroxynorzoanthamine (**3**).

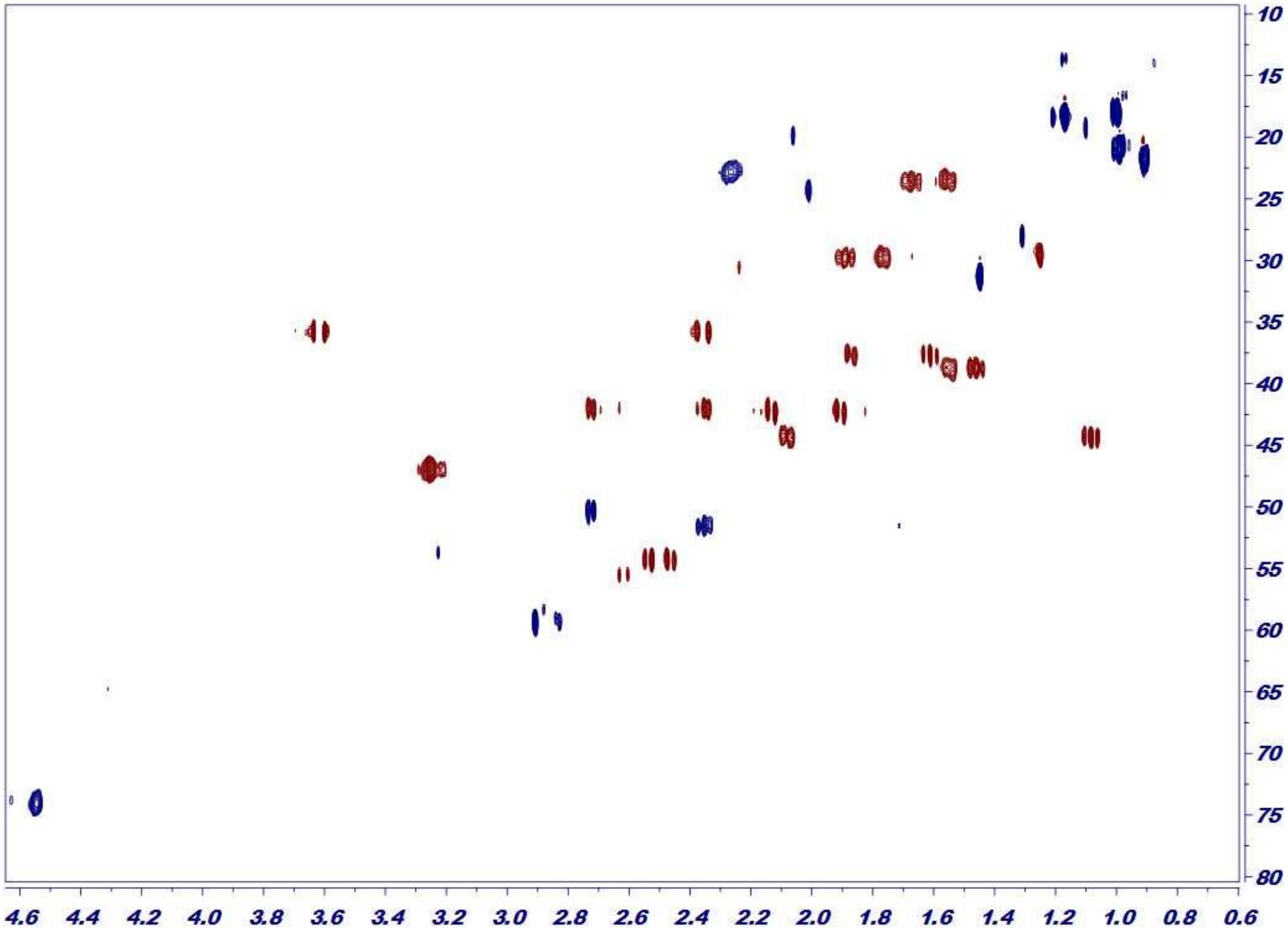

Figure S20. HMBC spectrum of 15-hydroxynorzoanthamine (**3**).

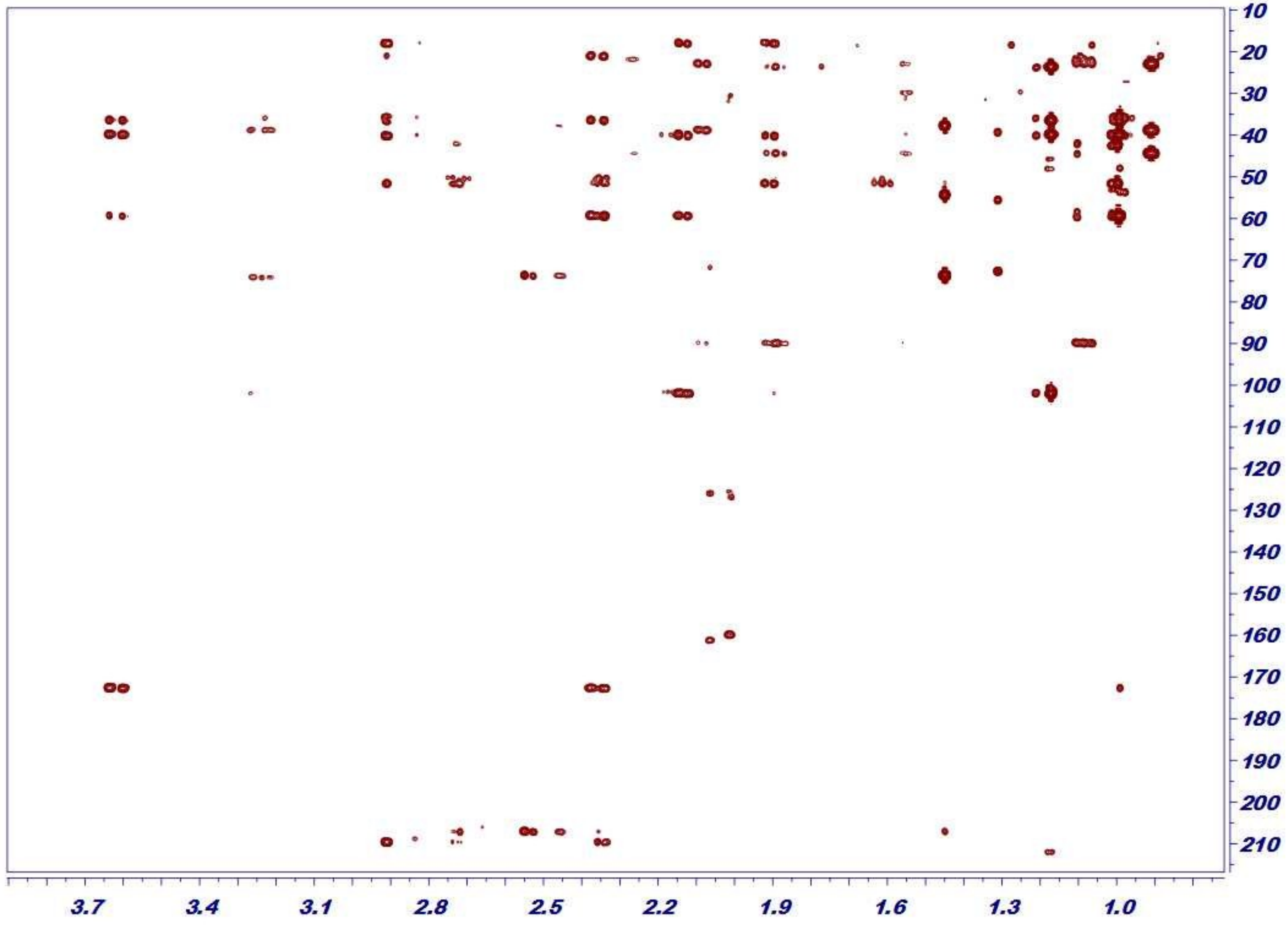

Figure S21. ROESY spectrum of 15-hydroxynorzoanthamine (**3**).

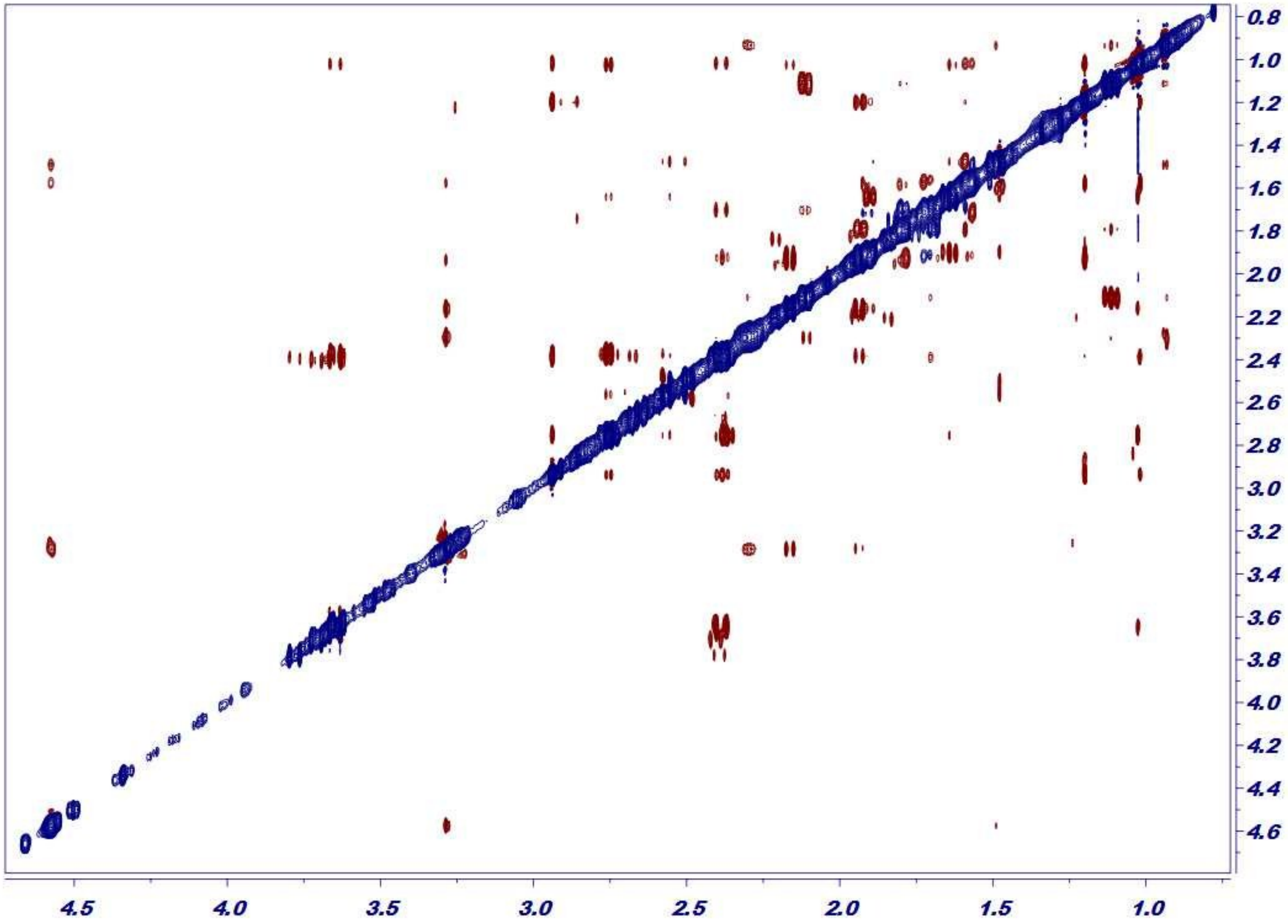

Figure S22. MS spectrum of 15-hydroxynorzoanthamine (**3**).

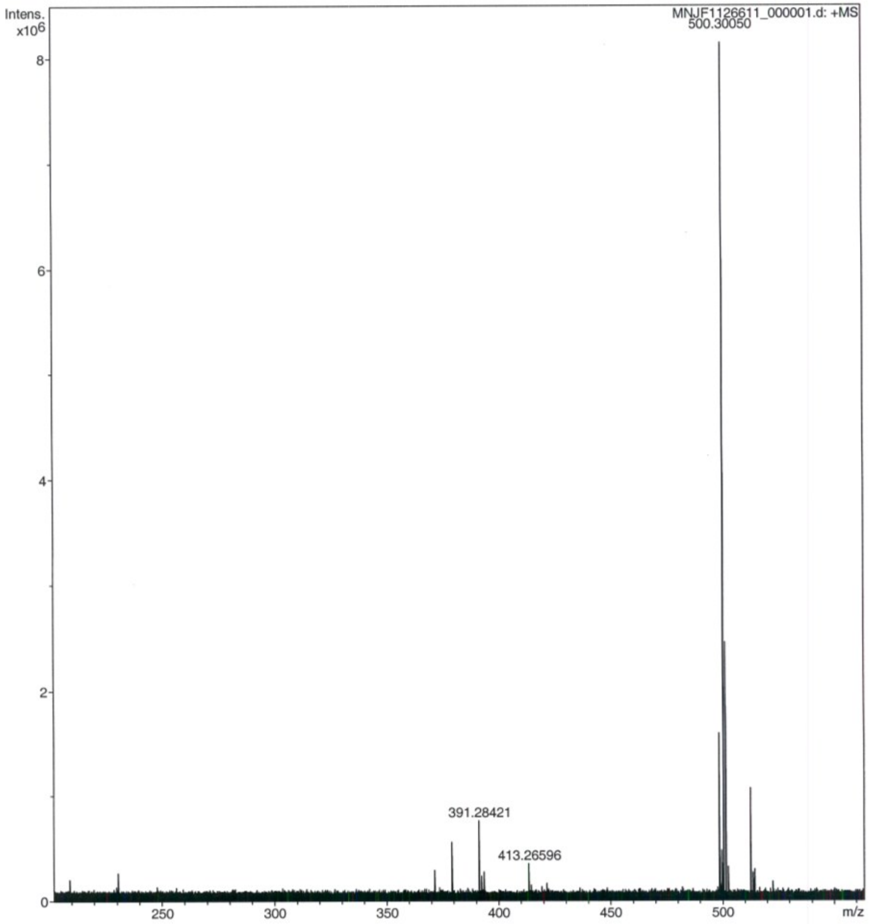

© 2014 by the authors; licensee MDPI, Basel, Switzerland. This article is an open access article distributed under the terms and conditions of the Creative Commons Attribution license (http://creativecommons.org/licenses/by/4.0/).
